# Whole-genome characterization of common rotavirus strains circulating in Vellore, India from 2002 to 2017: emergence of non-classical genomic constellations

**DOI:** 10.1186/s13099-023-00569-6

**Published:** 2023-09-20

**Authors:** Shainey Alokit Khakha, Tintu Varghese, Sidhartha Giri, Alan Durbin, Gene S. Tan, Maheswari Kalaivanan, Jasmin Helan Prasad, Gagandeep Kang

**Affiliations:** 1 The Wellcome Trust Research Laboratory, Division of Gastrointestinal Sciences, Christian Medical College, Vellore, India; 2 Department of Community Health, Christian Medical College, Vellore, India; 3 J. Craig Venter Institute, La Jolla, San Diego, CA 92037 USA; 4 Division of Infectious Diseases, Department of Medicine, University of California San Diego, La Jolla, San Diego, CA 92037 USA

**Keywords:** Rotavirus diarrhea, Whole genome sequencing, Rotavirus genotypes, Rotavirus diversity, Gene reassortment, Phylogenetics

## Abstract

Rotaviruses (RVs) are the most common etiological agent of acute gastroenteritis among young children, even after vaccine introduction in low-income countries. A whole-genome classification representing the 11 RV genes, was introduced for surveillance and characterization of RVs. This study characterized the common circulating strains in Vellore, India from 2002 to 2017 to understand rotavirus strain diversity and evolution using Whole genome sequencing (WGS) carried out on Illumina MiSeq. The 89% (92% of Wa-like, 86% of DS-1-like) of strains had classical constellations, while reassortant constellations were seen in 11% (8% of Wa-like, 14% of DS-1-like) of the strains. The rare E6-NSP4 in combination with DS-1 like G1P[8] and the emergence of the OP-354 subtype of P[8] were identified. Phylogenetics of RV strains revealed multiple subtypes circulating in the past 15 years, with strong evidence of animal to human gene transmission among several strains.

**Supplementary Information:**

The online version contains supplementary material available at 10.1186/s13099-023-00569-6.

## Introduction

Rotavirus A (RVA) is the most common cause of acute gastroenteritis in children less than five years of age and is responsible for approximately 128,500 annual deaths worldwide, mostly in low-middle income countries. India accounted for 21,357 deaths from the rotaviral disease in 2015, accounting for 20% of all global rotaviral deaths in children under five years of age [1, 2]. Rotavirus still remains the leading cause of diarrheal hospitalizations in low and middle income countries despite the large-scale use of rotavirus vaccines globally [3].

Rotaviruses have a three-layered structure with icosahedral symmetry, and have 11 segments of double-stranded RNA (dsRNA), which encode six structural proteins (VP1-VP4, VP6 and VP7) and six non-structural proteins (NSP1-NSP6) [4]. A binary classification has been widely used since the 1990s to classify RVA into G (VP7) and P (VP4) genotypes. Due to the segmented nature of the dsRNA genome, the genes encoding VP7 and VP4 can segregate independently leading to different combinations of G- and P- types [5]. Recently the binary classification system was extended to include the other nine genome segments based on nucleotide identity cut-off values to describe RVA strains and study RV diversity. The classification nomenclature for the structural and non-structural proteins of RVA is Gx-P[x]-Ix-Rx-Cx-Mx-Ax-Nx-Tx-Ex-Hx, representing VP7-VP4-VP6-VP1-VP2-VP3-NSP1-NSP2-NSP3-NSP4-NSP5 genes respectively, where x indicates the number of corresponding genotypes [6, 7]. At present, 42G, 58P, 32I, 28R, 24 C, 24 M, 39 A, 28 N, 28T, 32E and 28 H genotypes have been described [8]. The majority of human rotaviruses possess the Wa-like (Gx-P[x]-I1-R1-C1-M1-A1-N1-T1-E1-H1) constellation, of porcine origin, or the DS-1-like (Gx-P[x]-I2-R2-C2-M2-A2-N2-T2-E2-H2) constellation, that is of bovine origin [7]. In addition, a few strains belong to the AU-1-like (Gx-P[x]-I3-R3-C3-M3-A3-N3-T3-E3-H3) constellation which are of feline origin [6].

The whole genome characterisation of all 11 genes of RVA has provided valuable insights into RVA diversity that results from the accumulation of point mutations, genetic reassortment, and intragenic recombination [9–13]. In order to trace the evolutionary pattern of various strains, full genome characterization is required for interpretation of the origin of each segment of the RV genome [6]. Global studies on RVA strain surveillance and characterization have revealed high diversity of human RVA strains, including the possibility of gene reassortment between Wa-like and DS-1-like strains [14–19].

Surveillance from a single location over time can be valuable in understanding viral diversity and evolution. G1P[8] was the most predominant strain in India prior to vaccination (2012–2016), followed by G2P[4], G9 and G12 [20]. Vellore reported 32% of diarrheal hospitalizations due to RVA from 2005 to 2016. G2P[4] was the predominant strain in the initial years and was gradually replaced by G1P[8]. The emergence of G9P[4] replacing G9P[8] and emergence of G12 strains was also documented [21]. Molecular characterization of Indian strains has been performed mainly for the VP7 and VP4 genes, and data on the remaining nine genes are limited. In this study we report the whole genome analysis of common RVA strains circulating in Vellore, India, for the years 2002–2017.

## Results

### Sequence analysis and whole genome constellation determination

The majority of the G1P[8], G12P[6], G12P[8] and G9P[8] strains exhibited the classical Wa-like constellations, while the G2P[4] and G9P[4] genotypes exhibited the classical DS-1-like backbone (Table 1). A small proportion of reassortant strains were observed amongst the sequenced strains. A total of 88 G1P[8] strains genomes were sequenced, of which 82 (93%) had stable classical Wa-like constellation and 6 strains (7%) had reassortant constellations. Most G1P[8] reassortants were single or double reassortants with one or two gene segments inserted from DS-1 like viruses. Reassortment was observed in VP6, NSP1, NSP2, NSP3 and NSP4 genes. One strain (RVA/Human-wt/IND/TN020260/2017/G1P[8]) carrying a DS-1-like backbone had a rare E6 NSP4 gene (G1-P[8]-**I2-R2-C2-**Mx-**A2-N2-T2-E6*-*H2**). All G12P[8] (n = 12), G4P[6] (n = 1) and G4P[8] (n = 1) strains showed a classical Wa-like backbone, while 13 out of 16 (81%) G12P[6] rotaviruses were classical strains with Wa-like backbone and three (19%) were reassortants carrying a single reassortant gene. Three G9P[8] strains had the classical Wa-like backbone while one had a reassortant constellation with both Wa-like and DS-1-like profiles (G9-P[8]-**I2-R2-C2-**Mx-**A2-N2-**T1-E1-**H2).** 67 G2P[4] strains were sequenced of which 65 (96%) had stable classical DS-1 like backbone and three strains (4%) were reassortants. Reassortment was observed in VP3, NSP3, NSP4 and NSP5 genes. The strain designated RVA/Human-wt/IND/IN1004682_CMC_00026/2012/G2P[4] had the rare E6 reassortant gene in its backbone G2-P[4]-I2-Rx-C2-Mx-A2-N2-T2-**E6**-H2. Two of the three G9P[4] strains were single gene reassortants with E6-NSP4. The rare T6-NSP3 and H3-NSP5 reassortant genes were seen in the DS-1-like backbones of G6 and P[14] strains.

**Table 1:** Summary of the RVA strains sequenced from Vellore from year 2002 to 2017. Reassortant genes are marked in bold

Strain	Sequenced	Whole Genome constellation	No. of strains	Total	%	Typing Identification	% Reassortants in genogroups
G1P[8]	88	G1P[8]I1R1C1M1A1N1T1E1H1	82	82	93%	Classical Wa-like	8%
		G1P[8]**I2**R1C1MxA1N1T1E1H1	1	6	7%	Reassortant	
		G1P[8]I1R1C1M1A1N1T1E1**E2**H1	1				
		G1P[8]I1R1C1M1**A2**N1T1**T2**E1H1	1				
		G1P[8]**I2R2C2**Mx**A2N2T2E6H2**	1				
		G1P[8]I1R1C1A1**N2**T1E1H1	1				
		G1P[8]I1R1C1M1A1N1**T2**E1H1	1				
G12P[8]	12	G12P[8]I1R1C1M1A1N1T1E1H1	12	12	100%	Classical Wa-like	
G12P[4]	1	G12P[4]I1R1C1M1A1N1T1E1H1	1	1	-	Classical Wa-like	
G12P[6]	16	G12P[6]I1R1C1M1A1N1T1E1H1	13	13	81%	Classical Wa-like	
		G12P[6]I1**R2**C1M1A1N1T1E1H1	1	3	19%	Reassortant	
		G12P[6]I1R1C1**M2**A1N1T1E1H1	1				
		G12P[6]**I2**R1C1M1A1N1T1E1H1	1				
G9P[8]	4	G9P[8]I1RxI1M1A1N1T1E1H1	3	3	-	Classical Wa-like	
		G9P[8]**I2R2C2**Mx**A2N2**T1E1**H2**	1	1	-	Reassortant	
G4P[8]	1	G4P[8]I1R1I1M1A1N1T1E1H1	1	1	-	Classical Wa-like	
G4P[6]	1	G4P[6]I1R1I1M1AxN1T1E1H1	1	1	-	Classical Wa-like	
G2P[4]	67	G2P[4]I2R2C2M2A2N2T2E2H2	64	64	96%	Classical DS-1-like	14%
		G2P[4]I2RxC2MxA2N2**T1E1**H2	1	3	4%	Reassortant	
		G2P[4]I2R2C2**M1**A2N2**T1**T2**E1**E2**H1**	1				
		G2P[4]I2RxC2MxA2N2T2**E6**H2	1				
G2P[8]	2	G2**P**[8]I2R2C2M2A2N2T2E2H2	2	2	-	Reassortant	
G9P[4]	4	G9P[4]I2R2C2M2A2N2T2E2H2	1	1	-	Classical DS-1-like	
		G9P[4]I2R2C2M2A2N2T2**E6**H2	3	3	-	Reassortant	
G6	1	G6P[x]I2RxC2MxAxN2**T6**E2**H3**	1	1	-	Reassortant	
P[14]	2	GxP[14]I2R2C2M2AxN2**T6**E2**H3**	1	1	-	Reassortant	
		G8P[14]I2R2C2Mx**A11**N2**T6**E2**H3**	1	1	-	Reassortant	
**Total**	199	Note: 8 partially G or P typed strains were not included in this analysis					

### Distance analysis of individual gene segments

#### Diversity of Wa-like/DS-1-like strains

All the gene segments showed considerable diversity among Wa-like strains. Among the structural genes of Wa-like strains, the G12 and G1-VP7 gene segments showed the least diversity and among the non-structural genes, H1-NSP5 gene showed the least nucleotide diversity. Highest diversity was observed in P[8]-VP4 gene and A1-NSP1 gene (Table S5). On comparing the different genotypes of the same gene of Wa-like strains, it was observed that among the VP4 gene, the P[8]-genotype shows higher diversity than P[6]-genotype and among the VP7 gene, the G1-genotype shows higher diversity than the G12-genotype.

Among the structural genes of DS-1-like strains, VP4-P[4] showed the least diversity and VP3 gene showed the highest diversity. The NSP5 gene was the most conserved gene amongst the non-structural proteins and E2-NSP4 gene was the most diverse gene (Table S6). Overall, the G2P[4] showed higher diversity than G9P[4] strains across all the gene segments except for the VP7 gene which was more diverse among G9 than G2 strains.

#### Divergence of Wa-like/DS-1-like strains

Comparison of the study sequences with other RV strains sequences from GenBank clearly shows the divergence of gene sequences and continuous diversification over years. All the gene segments of the Wa-like strains from Vellore shared the least similarity at both NT and AA level with the human classical RV strains like Wa, KU, P, DC-2241, WI61 and ST3 which were circulating and characterized in 1970s (Table S7-S8). On the other hand, the study sequences shared more similarity with the classical RV strains like Dhaka-16, AM06-1 (Indian classical strain), Dhaka-12, Matlab-13, GER-172, Dhaka-25, B4633 and GER-172, which were isolated and characterized in the early 2000s. The highest similarities of the study strains were found with the strains from India reported previously (year 2000–2016). The study sequences shared very less similarity with the vaccine strain sequences like Rotarix, RotaTeq and 116E. Similarly, the DS-1-like study strains showed the least similarity with the DS-1 strain which is the primitive RV strain from 1970s and the strain 116E3D, which is a classical strain from India isolated in 1993 (Table S9). The study sequences shared a little higher similarity to the recently reported DS-1-like strains from Benin. The highest similarity was observed with the DS-1-like strains from India reported in past few years. Therefore, the majority of our study strains are human RV strains, which have diverged from the primitive RV strains circulating 50 years back around 1970s and have diversified its genome accumulating mutations over the years.

### Phylogenetic analysis

#### Analysis of VP7 gene

**Fig. 1:**
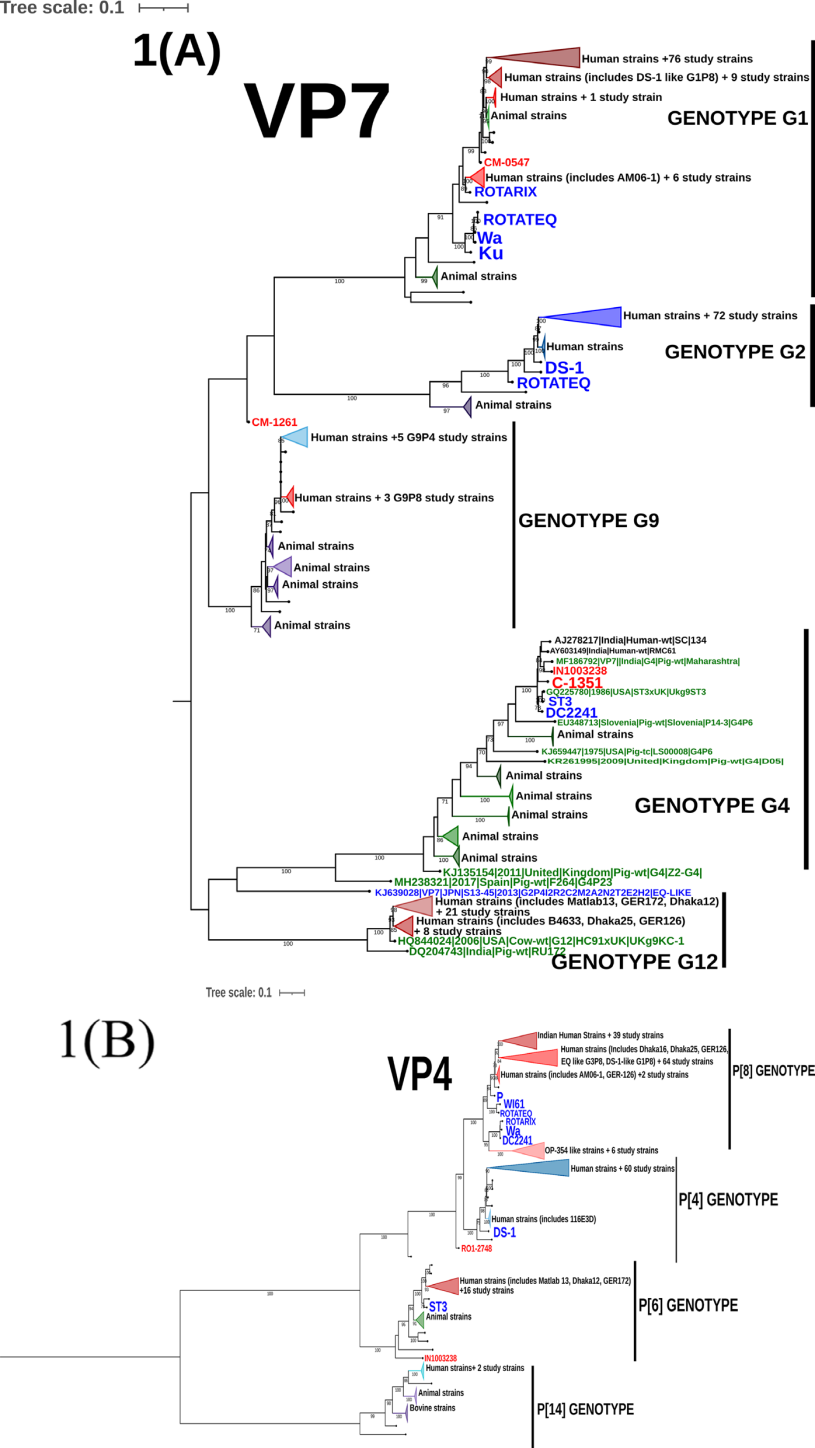

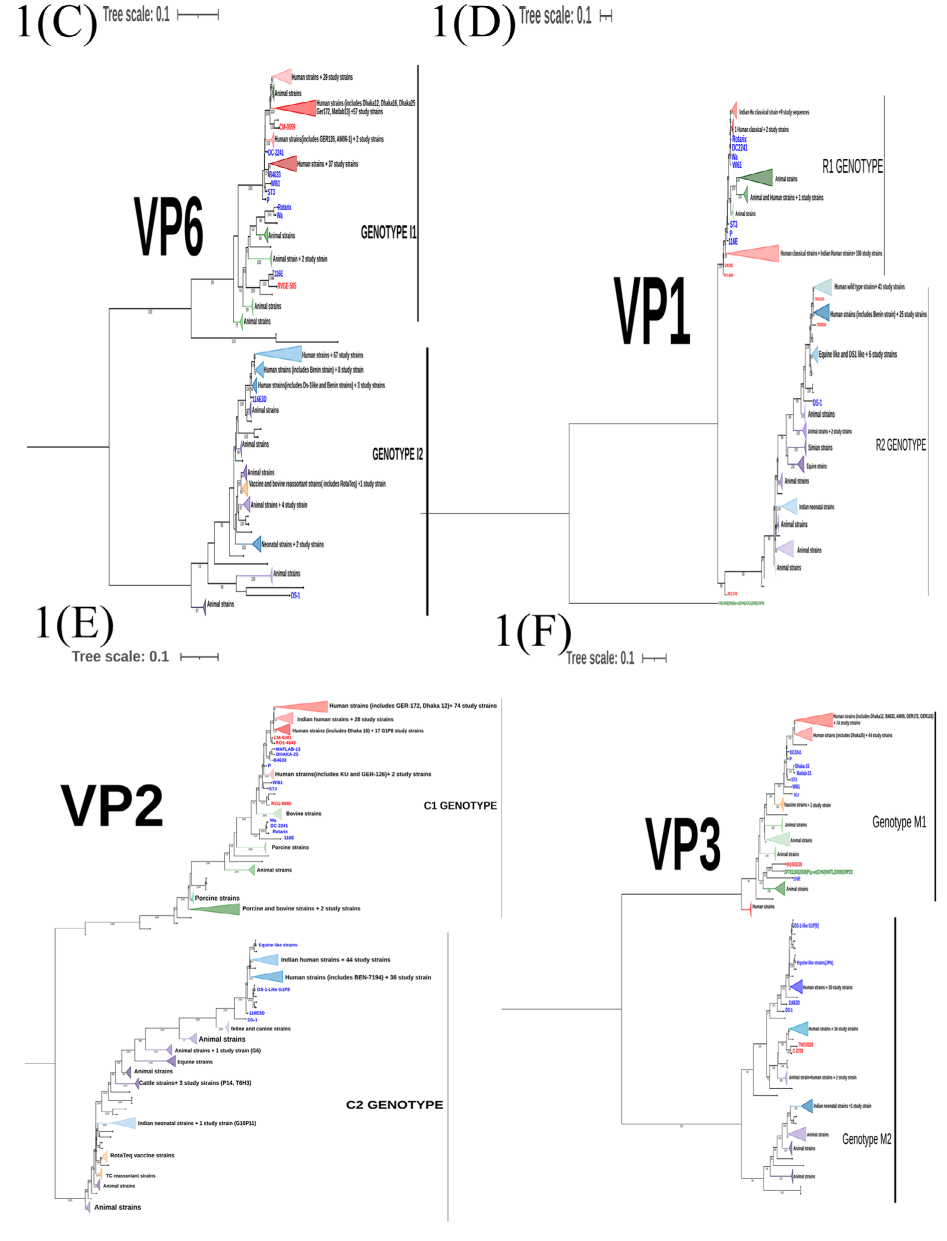

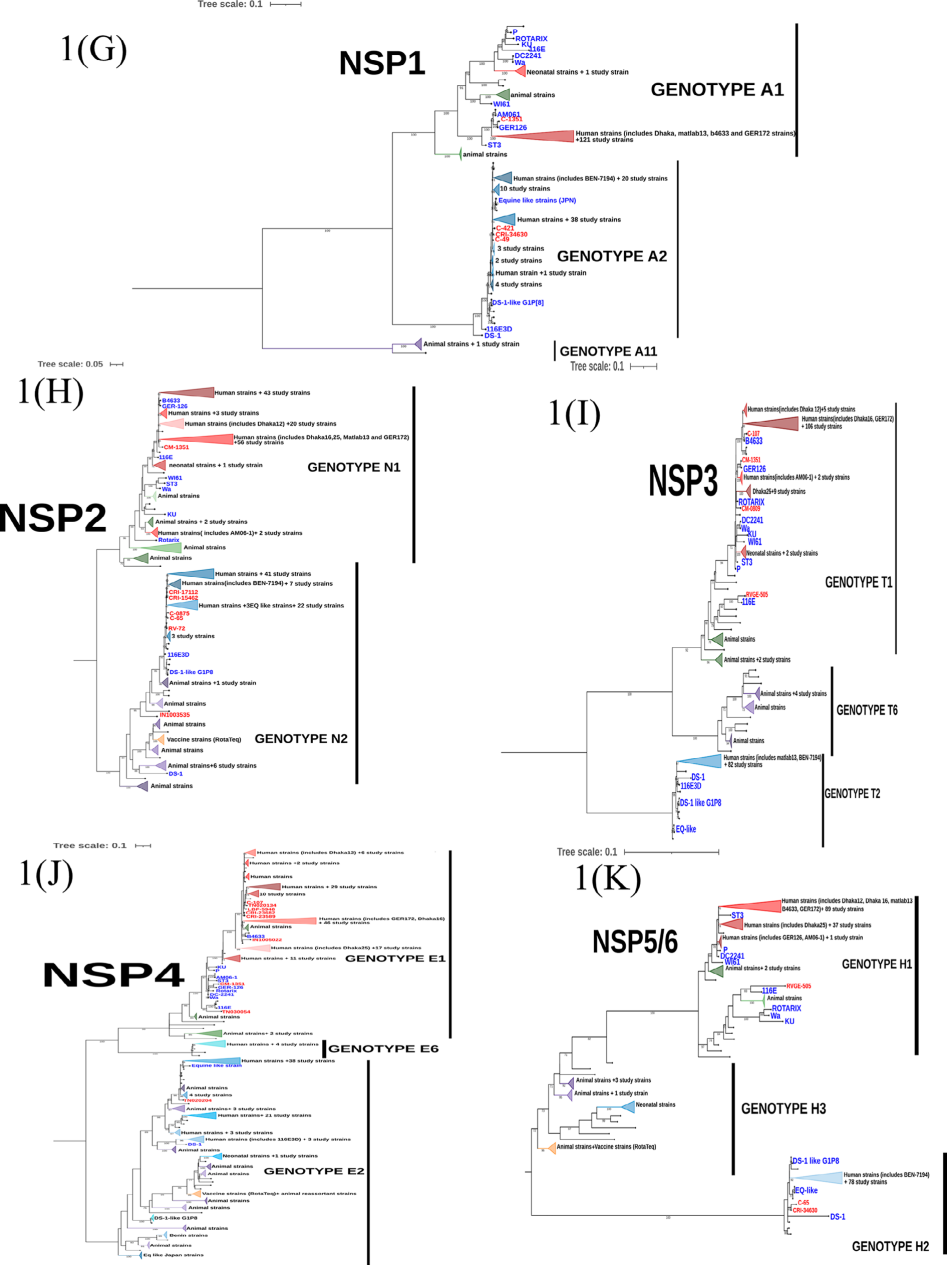
Maximum likelihood trees with bootstrap values for RVA VP7(A), VP4(B), VP6(C), VP1(D), VP2(E), VP3(F), NSP1(G), NSP2(H), NSP3(I), NSP4(J) and, NSP5/6(K) genes. The study sequences are coloured red, the reference classical human, equine-like, DS-1-like G1P[8] and vaccine sequences from GenBank are coloured blue, the animal sequences from GenBank are in green and the recent human sequences from India are in black. Phylogenetic clusters were collapsed for easy visualization and to make comprehensive trees. Clusters in shades of red represent RV gene sequences isolated from human hosts bearing Wa-like (genogroup 1) virus genotype, while clusters in shades of green represent RV gene sequences isolated from animal source bearing Wa-like virus genotype. Clusters in shades of blue represent RV gene sequences isolated from human hosts bearing DS-1-like (genogroup 2) virus genotype, while clusters in shades of purple represent RV gene sequences isolated from animal source bearing DS-1-like virus genotype. Vaccine and tissue culture reassortant clusters are highlighted in orange. Trees were drawn to scale as indicated by the scale bar which represents substitution per nucleotide site

**Fig. 2:**
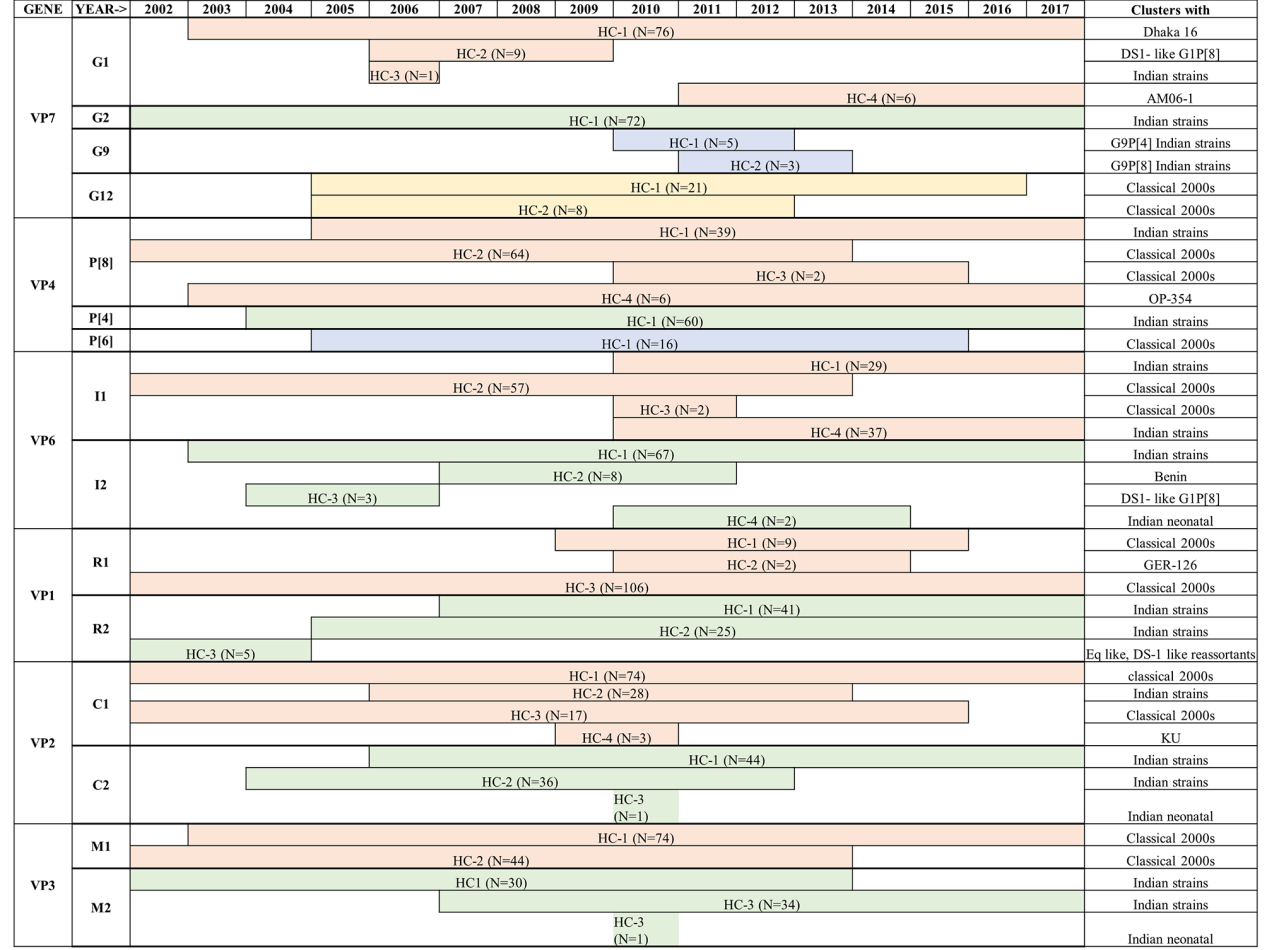
Different sub-genotype clusters observed within each RV genotype’s viral protein genes (HC-Human cluster, N-Number of strains in the cluster)

GTR + F + I + I + R4 substitution model was used to infer the VP7 gene tree (Fig. 1(A)). The G1 genotype sequences clustered into four different human clusters (HC-1 to HC-4) which represent the sub genotypes of the G1 genotype circulating in the study population (Fig. 2). Of these four different clusters, the human cluster 1(HC-1 N = 76) represents the major subtype which circulated in the past 15 years. This cluster share a close genetic similarity with Dhaka-16 classical strain from early 2000s. The other three human clusters were found occasionally with a low frequency. Only one study strain (RVA/Human-wt/IND/CM-0547/2013/G1P[8]) existed as a singlet branch.

The G2 genotype sequences formed only one human cluster representing single sub genotype circulating in the study population over the past 15 years. This is also supported by the high NT sequence similarity (96.75-100%) observed between G2 strains in the distance analysis.

The G9 study sequences form two distinct clusters where the five G9P[4] strains cluster separately from the three G9P[8] strains. A high divergence was also recorded in the distance analysis in concordance. The reassortant G9P[8] strain (RVA/Human-wt/IND/CM-1261/2015/G9P[8]-I2-R2-C2-Mx-A2-N2-T1-E1-H2) bearing both DS-1 and Wa like backbone lies as a singlet branch indicating high degree of genetic differences from the other human G9 sequences.

Two G4 strains share a close genetic similarity with the porcine RVA from India indicating animal to human transmission of the gene segment. These strains also clusters away from classical G4 strains (ST3 and DC2241).

G12 strains forms two distinct human clusters. The major cluster (HC-1) includes twenty-one G12 study strains which persisted over past 10 years. The minor cluster (HC-2) includes eight G12 strains circulating between 2005 and 2012 representing the sub-genotype that circulated in the early 2000s and diminished later.

#### Analysis of VP4 gene

Maximum Likelihood (ML) tree was built for VP4 gene sequences using the GTR + F + I + I + R4 substitution model (Fig. 1(B)). P[8] study sequences clustered into four human clusters (HC-1 to HC-4, Refer Fig. 2). The major cluster (HC-2) represents the sub-genotype that circulated from 2002 to 2013 and shared a close similarity with early 2000s RV classical strains. Reassortant G2P[8] strains (RVA/Human-wt/IND/IN1004655_CMC_00025/2012/G2P[8]-I2-RX-C2-MX-A2-N2-T2-E2-H2, RVA/Human-wt/IND/IN1005086_CMC_00027/2012/G2P[8]-I2-R2-C2-M2-A2-N2-T2-E2-H2) also clustered together in this branch putting forward evidence that the P[8] VP4 gene was probably introduced from the human Wa-like virus into the DS-1 backbone. The minor cluster HC-1 represent the current circulating sub-genotype of P[8] sequences that persistently circulated from 2005 to 2017. The other two clusters of P[8] were observed occasionally in which the HC-4 cluster includes six study strains identified between 2003 and 2017 clustering closely with OP-354 like P[8] sequences which has been reported for rapid spread across the globe [22].

All the P[4] sequences clustered into one cluster (HC-1) and show a separate branch away from the early classical RV strains like DS-1 and 116E3D. A single strain (RVA/Human-wt/IND/RO1-2748/2010/G12P[4]) with a Wa-like backbone lies as a singlet branch.

The P[6] sequences formed a monophyletic cluster and shared close similarity with that of classical G12P[6] strains- Matlab13, Dhaka12 and GER172 from 2000s. The single G4P[6] strain (RVA/Human-wt/IND/IN1003238_CMC_00038/2011/G4P[6]) stays as a singlet branch away from the other P[6] sequences.

Two P[14] sequences clustered with other two Indian human strains reported from 2014 (G10P[14]) and share close branches with animal sequences. This indicates P[14] had originally evolved from animals and is now reported from humans across globe in combination with different G types.

#### Analysis of VP6, VP1-VP3 genes

A maximum likelihood tree for VP6, VP1, VP2 and VP3 gene (Fig. 1(C-F)) were built using GTR + F + I + I + R4/R5 substitution model. These viral protein genes sequences showed multiple sub genotype clusters for genogroup1 (I1, R1, C1 and M1) and genogroup 2 (I2, R2, C2 and M2) as shown in Fig. 2. Three major human clusters were observed in VP6-I1 of which HC-2 shared close similarity with 2000s classical RV strains and persisted in our setting from 2002 to 2013. HC-1 and HC-4 represent the recent circulating strains which has diverged considerably from the 1970 and 2000 s classical RV strains. The VP1-R1 sequences showed one major cluster (HC-3) that persisted throughout the study period and shared close similarity with 2000s classical RV strains. The other two sub-genotype clusters were found occasionally. The VP2-C1 sequences showed one major (HC-1), two minor (HC-2 & HC-3) and one occasionally occurring sub-genotypic clusters. The major cluster persisted throughout the study period sharing close similarity with classical 2000s RVs strains. The minor clusters declined towards 2013 to 2015 and it is important to note that the minor cluster (HC-3) includes gene sequences specifically from only G1P[8] specificity. VP3-M1 sequences clustered into one major cluster which persisted throughout and one minor cluster which persisted till 2013.

Looking at the structural genes of DS-1 like viruses, the VP6-I2 sequences showed one major sub-genotypic cluster and two occasionally occurring clusters. One minor cluster includes two study strains (one G10P[11] and a reassortant G12P[6]) which clustered with neonatal I2 human sequences reported previously from India [23]. This emphasizes that neonatal I2-VP6 sequences vary significantly from other human I2-VP6 sequences. The VP1-R2 gene sequences resolve into two major sub-genotypic cluster (HC-1 & HC-2), which share close similarity with previously reported Indian sequences and persists till date. One rarely occurring sub-genotype cluster was seen during the first couple of years which shares close similarity with R2 sequences from reassortant equine like strains and DS-1 like G1P[8] strains. Two major and one neonatal sub-genotypic cluster was observed among the VP2-C2 gene sequences. VP3-M2 sequences clustered into two-major and one neonatal sub-genotypic cluster. The major cluster HC-1 continued to persist from beginning till 2017 while the other major cluster (HC-2) was found only till 2013.

#### Analysis of non-structural proteins (NSPs) genes

**Fig. 3:**
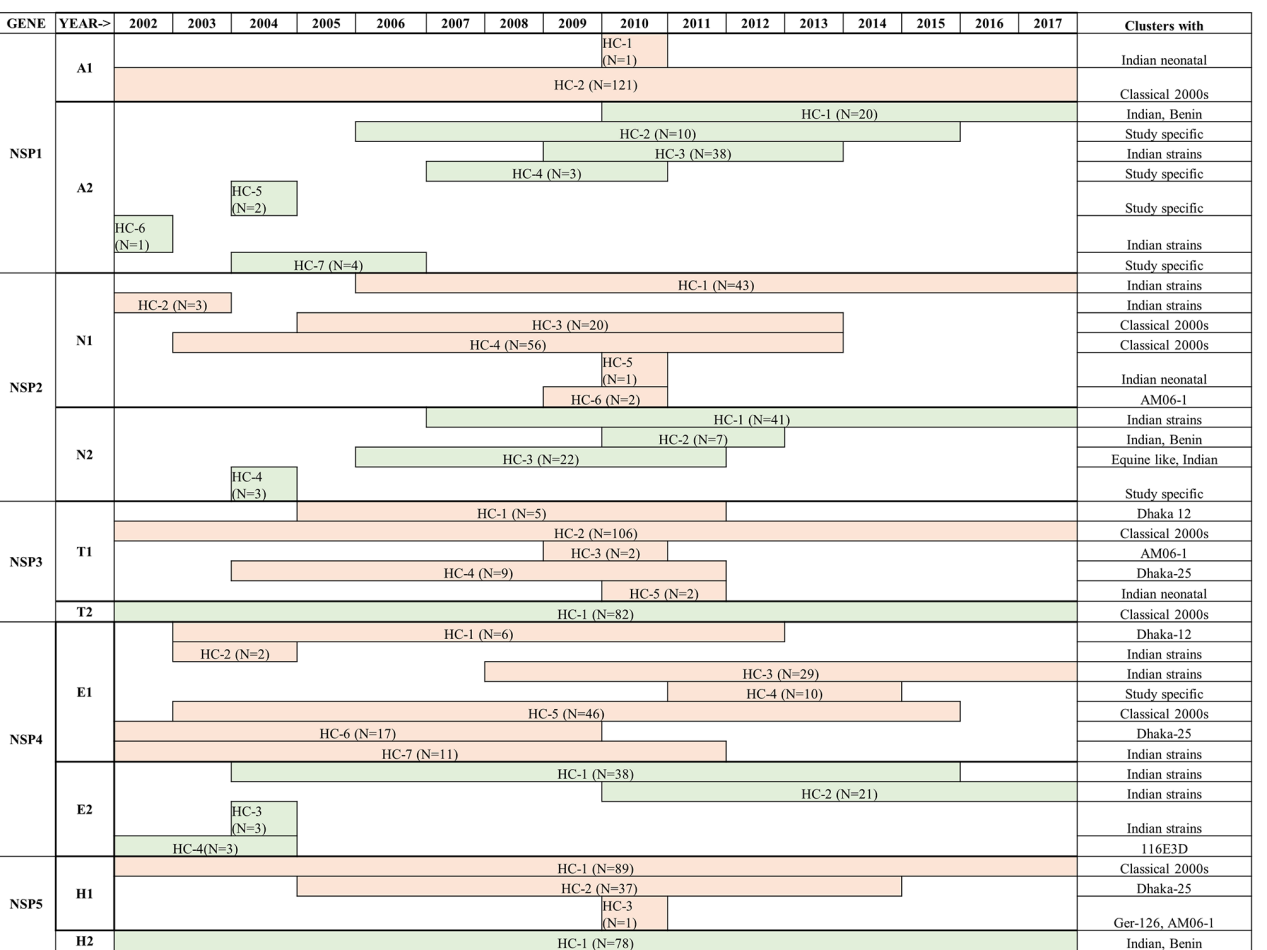
Different sub-genotype clusters observed within each RV genotype’s non-structural protein genes (HC-Human cluster, N-Number of strains in the cluster)

Diversity of NSP gene sequences varied with the genogroup (Refer Fig. 1(G-K) and Fig. 3). Among the Wa-like viruses (Genogroup I), NSP1-A1 gene appeared conserved while the other NSPs were diverse showing multiple sub-genotypic clusters of which the NSP4-E1 gene showed the highest diversity. On the other hand, among the DS-1 like viruses (Genogroup II), NSP3 and NSP5 genes were conserved, while the NSP1, NSP2 and NSP4 gene had multiple sub genotype clusters. NSP1-A2 gene showed the highest diversity among the Wa -like viruses.

Only one major sub-genotypic cluster and one neonatal cluster was observed in NSP1-A1 gene sequences, where the major cluster persisted throughout the study period. NSP2-N1 gene sequences were diverse and segregated into two major (HC-1 & HC-4), one minor (HC-3), two occasional (HC-2 & HC-6), and one neonatal (HC-5) sub-genotypic clusters. HC-4 and HC-3 represent the sub-genotype which circulated until 2013 while, HC-1 represent the current circulating sub-genotype. NSP3-T1 gene sequences resolved into one major (HC-2), three occasional and one neonatal (HC-5) sub-genotype clusters. NSP4-E1 gene sequences showed the maximum sub-genotypic clusters with one major (HC-5), two minor (HC-3 & HC-6) and four occasional clusters. None of the sub-genotype clusters seem to persist throughout the study period. The NSP5-H1 gene sequences show one major, one minor and one occasional sub-genotypic clusters. The major cluster persisted throughout the past 15 years while the minor cluster persisted from 2005 to 2014.

NSP1-A2 gene sequences segregates into one major (HC-3), one minor (HC-1) and five occasional sub-genotypic clusters. In the beginning, occasional sub-genotypes were in circulation the major sub-type (HC-3) dominated from 2009 but declined by 2013. Currently, the HC-1 sub-genotype of A2 genotype persists among DS-1 like strains. HC-2, HC-4, HC-5 and HC-7 clusters represent Vellore-specific sub-genotypes. One major (HC-1), one minor (HC-3) and two occasional sub-genotypic clusters were observed in NSP-N2 gene sequences. The major cluster persisted in circulation since 2007 and represent the recent circulating sub-genotype. The minor cluster, which was in circulation from 2006 to 2011, shares a high similarity with equine-human reassortant RV strains reported from Japan. HC-4 represent the Vellore-specific sub-genotype. NSP3-T2 and NSP5-H2 gene sequences showed only one sub-genotype in circulation in past 15 years. NSP4 gene sequences shows one major, one minor and two occasional sub-genotypic clusters, where the major cluster persisted till 2015 and the minor cluster appeared in 2010 and represents the currently circulating sub-genotype.

#### Inter-species transmission

Animal derived RV backbone and genes were seen in a few of our study strains details of which is shown in Table 2. Six of the study strains bearing atypical G and P type showed multiple genes that clustered closely to the animal RV sequences. Of these, one strain (G4P[6] genotype) was a porcine like strain circulating in human hosts with most of the genes of porcine origin. Another six strains bearing common genotypes showed single-reassortant gene of animal origin in their genetic backbone. Genome constellation of these reassortants is mentioned in Table 2. Pigs and cattle RV were the most common source of animal reassortant strains in our setting. The genotypes G6, P[14], A11, T6 and H3 were associated specifically with animal-human reassortant RVs. Among the common genotypes (G1P[8] and G2P[4]) only single gene reassortment was reported. Though the genotype constellation of these common strains appeared like a stable backbone, analysis of single gene sequences by the in depth molecular phylogenetics revealed the presence of rare animal-human reassortant genes. One G1P[8] strain(RVA/Human-wt/IND/CM-0059/2012/G1P[8]) carried VP6 gene of a bovine origin. One G2P[4] strain (RVA/Human-wt/IND/C-65/2006/G2P[4]) carried single NSP4 gene of bovine source and four G2P[4] strains carried NSP2 gene which had originated from cattle strains. Presence of such population of reassortant strains reflects the potency of these strains to expand its host and cause symptomatic disease warranting regular monitoring of circulating RVA strains.

#### Vaccine reassortants

In-depth molecular phylogenetics of genes revealed that few RV vaccine reassortant strains circulated in our setting (Table 2). Two RotaTeq (RV5) vaccine reassortant strains were detected with single reassortant gene. Reassortment was seen in VP3 gene of a G4P[8] strain and VP6 gene of a G1P[8] strain. A G12P[6] strain RVA/Human-wt/IND/RVGE-505/2012/G12P[6] was detected with three reassortant genes-VP6, NSP3 and NSP5 from 116E natural reassortant neonatal vaccine strain. The rest of its gene shared high similarity with neonatal strains from India. Detection of vaccine reassortants in our setting is an indication of multiple complex reassortment events happening between vaccine strains and human strains during mixed infections and the ability of these new reassortants to persist and circulate through natural selection.

**Table 2:** List of vaccine reassortants and animal reassortants detected in the study. Respective reassortant genes are highlighted in bold. AN-Animal gene, Po-Porcine gene, Bo-Bovine gene, Cap-Caprine gene, RV5-Rotateq strain gene, 116E- Rotavac strain gene, H-hospitalized case, C-non-hospitalized community case, NA-data not available

	Host details				
Vaccine reassortants	Age(Months)	Sex	Setting	Genome constellation	
RVA/Human-wt/IND/RVGE-505/2012/G12P[6]	NA	NA	H	G12P[6]-**I1(116E)**-R1-C1-M1-A1-N1-**T1(116E)**-E1-**H1(116E)**	
RVA/Human-wt/IND/C-1351/2010/G4P[8]	11	F	H	G4P[8]-I1-R1-C1-**M1(RV5)**-A1-N1-T1-E1-H1	
RVA/Human-wt/IND/IRID-2631/2009/G1P[8]	NA	NA	C	G1P[8]-**I2(RV5)**-R1-C1-Mx-A1-N1-T1-E1-H1	
**Animal reassortants**				**Genome constellation**	
RVA/Human-wt/IND/IN1003238_CMC_00038/2011/G4P[6]	10	M	C	**G4(Po)**P[6]-**I1(Po)-R1(Po)-C1(Po)-M1(Po)**-Ax-**N1(Po)-T1(Po)-E1(Po)-H1(Po)**	
RVA/Human-wt/IND/IN1004413_CMC_00022/2012/GxP[14]	15	M	C	GxP[14]**-I2(Bo)-R2(Bo)-C2(Bo)-M2(Bo)**-Ax-**N2(Bo)-T6(Bo)-E2(Bo)-H3(Bo)**	
RVA/Human-wt/IND/TN020204/2017/G8P[14]	00	M	H	G8P[14]-**I2(Bo)**-R2-**C2(Bo)**-Mx-**A11(Bo/Cap)-N2(Bo)-T6(Bo)-**E2-**H3(Bo)**	
RVA/Human-wt/IND/RO1-14518/2010/G2P[4] + P[8]	NA	NA	C	G2P[4] + P[8]-**I2(Bo)-R2(Bo)-C2(Bo)-M2(Bo)-**A2**-N2(Bo)-**T2**-T6(Bo)-**E2**-**H2**-H3(Bo)**	
RVA/Human-wt/IND/IN1003535/2011/G6P[x]	08	F	C	G6P[x]-**I2(Bo)**-Rx-**C2(Bo/Cap)**-Mx-Ax-**N2(AN)-T6(Bo)-E2(Bo)-H3(Bo)**	
RVA/Human-wt/IND/IN1000458_CMC_00052/2010/GXPX	06	M	C	GxP[x]-**I1(Po)**-Rx-**C1(Po)**-Mx-Ax-**N1(Po)-T1(Po)-E1(Po)-H1(Po)**	
RVA/Human-wt/IND/CM-0059/2012/G1P[8]	18	F	H	G1P[8]- **I1(Bo)**-R1-C1-M1-A1-N1-T1-E1-H1	
RVA/Human-wt/IND/C-65/2006/G2P[4]	15	F	H	G2P[4]-I2-R2-C2-M2-A2-N2-T2-**E2(Cap)**-H2	
RVA/Human-wt/IND/CM-0180/2013/G2P[4]	07	M	H	G2P[4]-I2-R2-C2-M2-A2-**N2(Bo)**-T2-E2-H2	
RVA/Human-wt/IND/CM-0170/2012/G2P[4]	04	M	H	G2P[4]-I2-R2-C2-M2-A2-**N2(Bo)**-T2-E2-H2	
RVA/Human-wt/IND/CM-0423/2013/G2P[4]	32	F	H	G2P[4]-I2-R2-C2-M2-A2-**N2(Bo)**-T2-E2-H2	
RVA/Human-wt/IND/CM-0002/2012/G2P[4]	13	M	H	G2P[4]-I2-R2-C2-M2-A2-**N2(Bo)**-T2-E2-H2	

## Discussion

This study reports on 199 complete genome sequences of common RV strains from Vellore, India, selected over a period of 15 years (2002–2017). This new data will significantly increase the number of publicly available whole genome sequences from Indian settings. Genotypes G1P[8], G2P[4] and G12P[6]/P[8] are detected at high frequencies in India [24–32] and also other parts of the world [33–37]. Eighty-eight G1P[8], thirty G12 and sixty-seven G2P[4] strains were sequenced and characterized along with few other strains in small numbers. It is important to note that the majority of strains had a classical Wa like (92%) and DS-1-like backbones (86%), while few strains (8% of Wa and 14% of DS-like) bore a reassortant’s backbone. Amongst the G1P[8] strains 7% were reassortant, while 4% of G2P[4] strains 4% were reassortant. Similar data was reported from studies conducted in United states and Africa where majority of the G1P[8] and G2P[4] sequences had the consensus Wa and DS-1 like backbone and very less proportion of reassortants [38–42]. This also supports the fact that these common circulating viruses carry a very stable backbone and the genotype 1 is linked with G1 specificity and genotype 2 is linked to G2 specificity in majority of the cases and reassortment is a rare event. The reassortant strains had one to nine reassortant genes. One DS-1 like G1P[8] strain (RVA/Human-wt/IND/TN020260/2017/G1P[8]) was detected with rare E6-NSP3 (G1P[8]-I2-R2-C2-Mx-A2-N2-T2-E6-H2) and appear to have originated from 3 to 4 reassortment events between human DS-1-like and G2P[4] and Wa like G1P[8] strains. DS-1 like G1P[8] was first reported from South Africa in 2008 and had later been reported from various studies from Vietnam, Philippines, Japan, United States and Malawi [15, 18, 39, 43, 44]. DS-1 like G1P[8] in combination with E6 genotype is reported for the first time from an Indian setting. It is noteworthy that, amongst the G12 strains, G12P[8] had no reassortant strains while amongst G12P[6] strains there were 19% reassortant strains. Among the few sequenced G9 (N = 9) strains, 44% were reassortant. Unusual reassortant G9 strain- RVA/Human-wt/IND/CM-1261/2015/G9P[8] was identified with the constellation G9P[8]-I2-R2-C2-Mx-A2-N2-T1-E1-H2 where six genes appear to have reassorted from a DS-1 like strain into a Wa like G9P[8] strain. Three atypical G9 strains from 2011 with G9P[4]I2-R2-C2-M2-A2-N2-T2-E6-H2 constellation was observed with rare E6-NSP4 gene. Similar reassortant strains of G9P[4] strains had been previously reported from India, Latin America and recently from Benin in 2016 [41, 45–47]. In our study the rare E6 genotype was also observed with G1P[8] and G2P[4] strains during 2017 and 2012 respectively. The E6-NSP4 was first reported from Bangladesh in combination with G12P[6] in 2000 [37]. During the same year, it was reported from New Delhi, India in combination with G8P[6] [48]. Later in 2017, it was reported with G2P[4] strains from Pune, India, in samples from 2009 to 2013 [49]. E6-NSP4 has now been reported only from the human hosts and is emerging in different geographical locations among the DS-1 like strains mostly and Wa-like strains in rare occasions. Unusual reassortant strains characterized in this study also includes two strains- (RVA/Human-wt/IND/IN1004655_CMC_00025/2012/G2P[8] and RVA/Human-wt/IND/IN1005086_CMC_00027/2012/G2P[8]) with genetic constellation G2P[8]-I2-R2-C2-M2-A2-N2-T2-E2-H2 detected in 2012 which appears to be VP4 reassortant between Wa-like and DS-1-like RV. Strains with similar reassortant constellation was identified previously in USA [39].

Few vaccine reassortants were also detected in our setting. A RotaTeq (RV5) vaccine VP6 reassortant strain-RVA/Human-wt/IND/IRID-2631/2009/G1P[8] was identified with constellation G1(WT)P[8](WT)-I2(RV5)-R1(WT)-C1(WT)-Mx-A1(WT)-NI(WT)-T1(WT)-E1(WT)-H1(WT). VP6 gene from RV5 strain could have reassorted into Wa-like strain. Another RotaTeq derived human strain (RVA/Human-wt/IND/C-1351/2010/G4P[8]) was identified with the constellation G4(WT)P[8] (WT)-I1(WT)-R1(WT)-C1(WT)-M1(RV5)-A1(WT)-N1(WT)-T1(WT)-E1(WT)-H1(WT), where M1-VP3 gene appear to be of vaccine origin which reassorted with Wa-like strain. RotaTeq was not included in the national immunization program at this time but was licensed for private use. It is quite possible that shed vaccine strains could circulate and reassort. It is important to note that such strain appear like a classical Wa-like RV from its constellations, but the in-depth whole genome analysis reveals the true origin of the strain. Hence whole genome analysis becomes an important tool to fully characterize and understand the true origin of the circulating strains. This observation is consistent with the findings of another research group from Brazil, Nicaragua and United States who have reported that such reassortment events are expected considering attenuation of RotaTeq vaccine and the segmented nature of RVA genome [50–53]. Vaccine reassortant strain with partial Rotarix and partial RotaTeq genes in backbone was previously reported from USA [39]. Triple reassortant strain RVA/Human-wt/IND/RVGE-505/2012/G12P[6] was identified with constellation G12P[6]-I1-R1-C1-M1-A1-N1-T1-E1-H1 where the VP6, NSP3 and NSP5 gene clustered closely to the 116E strain and rest of the genes clustered with other Indian neonatal strains. 116E is a natural reassortant neonatal RV strain that was identified in 1985 in New Delhi, India, and was later developed into Rotavac vaccine strain [54]. It was licensed in 2014 and included in National Immunization programme in 2016. Our strain was isolated in 2012 prior to licensure and introduction of 116E vaccine. Therefore, this strain does not appear to be of vaccine origin instead it could have originated from 116E like wildtype strains which could have been circulating at that time.

Rotaviruses are generally species specific but cross species transmission is possible and has been demonstrated frequently. Surveillance of circulating rotaviruses in the human population has revealed the presence of several uncommon genotypes. Many of these have been found in animals, and it is possible that they arose in the human population through zoonotic transmission [55–58]. Six study strains showed multiple genes of animal origin in its backbone and six other strains with single gene reassortment from an animal source. Strain RVA/Human-wt/IND/IN1003238_CMC_00038/2011/G4P[6] with genetic constellation G4(AN)P[6]-I1(AN)-R1(AN)-C1(AN)-M1(AN)-Ax-N1(AN)-T1(AN)-E1(AN)-H1(AN) appears to be a human-porcine reassortant strain with majority of the genes clustering with porcine strain. Two P[14] strains with unusual T6-NSP3 and H3-NSP4 RVA/Human-wt/IND/**IN1004413**_CMC_00022/2012/GxP[14], and RVA/Human-wt/IND/**TN020204**/2017/G8P[14] shows constellation GxP[14]-I2(AN)-R2(AN)-C2(AN)-M2(AN)-Ax-N2(AN)-T6(AN)-E2(HU)-H3(AN) and G8P[14]-I2(AN)-R2(HU)-C2(AN)-Mx-A11(AN)-N2(AN)-T6(AN)-E2(HU)-H3(AN) respectively. Both the P[14] appears to be human animal reassortant. It is also noteworthy that these strains carry the unusual T6-NSP3 and H3-NSP5 genes which are mostly seen in animal rotaviruses. Several P[14] strains with DS-1 like backbone bearing T6-NSP3, H3-NSP2 and A11-NSP1 gene has previously been characterized from humans and were closely related to RV strains from sheep, goats and cattle [59]. Two other strains RVA/Human-wt/IND/RO1-14518/2010/G2P[4] + P[8] with constellation G2P[4] + P[8]I2(AN)-R2(AN)-C2(AN)-M2(AN)-A2(HU)-N2(AN)-T2(HU)T6(AN)-E2(HU)-H2(HU)H3(AN)) and RVA/Human-wt/IND/IN1003535_CMC_00014/2011/G6P[x] with constellation G6P[x]-I2(AN)-Rx-C2(AN)-Mx-Ax-N2-T6(AN)-E2(AN)-H3(AN) shows multiple genes from animal origin including the unusual T6-NSP3 and H3-NSP5. G6 in combination with P[1], P[5], P[7] and P[14] strains are mostly of animal origin and have been reported from cow, sheep, antelopes and horses previously [60]. Hence, our G6 study strain proves to be a pure animal RV strain transmitted to human host. An untyped Wa like strain RVA/Human-wt/IND/IN1000458_CMC_00052/2010/GXPX appears to be porcine derived RV with constellation GxP[x]-I1(AN)-RX-C1(AN)-MX-AX-N1(AN)-T1(AN)-E1(AN)-H1(AN) where the six characterized genes were of porcine origin. Four strains of typical DS-1 like backbone (CM-0180, CM-0170, CM-0423 and CM-0002) with single N2-NSP2 animal gene reassortment were detected. One strain (C-65) of typical DS-1 like backbone had single E2-NSP4 animal reassortant gene. Strain (CM-0059) of typical Wa like constellation also had single I2-VP6 animal reassortant gene. This suggests that not just unusual genotype but common genotypes with classical constellation houses genes of animal origin as a result of reassortment which goes undetected in genotyping assays and is evident only when sequences are subjected to phylogenetic analysis.

Gene specific analyses of the sequenced strains clearly reflects the heterogeneity and diversity among each genotype as indicated by multiple clusters of sequences for each gene. Among the Wa like strains the VP7, VP4, VP1, VP3, NSP1 and NSP5/6 genes appear less diverse than VP6, VP2, NSP2, NSP3 and NSP4 genes. While among the DS-1 like strains the VP4, VP7, VP1, VP3, NSP3 and NSP5 genes appear less diverse than VP6, VP2, NSP1, NSP2 and NSP4 genes. DS-1 like strain’s G2-VP7, P[4]-VP4, T2-NSP3 and H2-NSP5 genotypes showed only one sub-genotype circulating in the past 15 years suggesting these genotypes to be highly conserved. While genes of other genotypes showed at least two different circulating sub-genotypes. Amongst the Wa-like strain genotypes, only A1-NSP1 gene appeared to be highly conserved with only one human sub-genotype circulating in the past 15 years. Previous comparative studies have reported circulation of multiple sub-genotypes referred as alleles for each RVA gene which also differ at different geographical settings [38–42]. Each RV gene and genotype has varying mutation/substitution rates, as reported by some studies [12, 61–65]. This could be one the reasons for varying diversity observed for different RV gene or genotype. It is important to note that six strains with OP-354 like P[8]-VP7 sequences were circulating in our setting. OP-354 like P[8], the new divergent form of P[8] strains, originated in south east Asia and has rapidly spread across the continent [22]. OP-354 like P[8] are known to exist with various G types. In our study, OP-354 like P[8] were seen in combination with G1 and G9 strains. There have been few reports which suggest that OP-354 P[8] are associated with severe forms of diarrhea [66, 67] and in future it would be interesting to analyse such association in our setting. Equine like rotavirus strains are now being reported from various studies where equine like DS-1 backbone has been observed in combination with G3P[8], G3P[6] and G3P[4] [68–70]. In our study, five equine like R2-VP1 sequences and 22 equine-like N2-NSP2 sequences were detected in G2P[4] strains. This result is quite unusual and new G2P[4] strains with equine like genes have not been reported earlier. There could be a possibility that these VP1 and NSP2 genes could have reassorted from equine like human strains however, more analysis would be required to support the data.

Our study has a few limitations since we were not able to sequence all the strains isolated during 2002–2017 and only few strains from each year were randomly selected for characterization. Other important prevalent strains like G3P[8], G1P[6] and G9P[8]/P[4] were not included whose genetic surveillance is equally important. Very few unusual strains (where evidence of reassortments and zoonotic transmission are more likely) were sequenced and there are chances that characterizing more of such strains will add to the knowledge on rotavirus diversity in Indian settings. For some of the study strains, few genes failed to be sequenced (specially for the unusual strains) and better sequencing protocols are required to meet such challenges.

## Conclusion

To summarize, this study found that majority of Wa and DS-1 like strains in our setting carry a stable genetic constellation but the individual genes keep evolving into new sub-genotypes. All the genes are prone to occasional reassortment events and emergence of mixed genetic constellation, though the chances are less than 10%. Some genes have proved to be very conserved in the past years, while few other genes have diverged significantly to create new sub-genotypes. Our study provides the baseline whole-genome sequence data on the common circulating strains in India from the pre-vaccine period which will be crucial to study the evolution of RVA strains since large scale use of rotavirus vaccines especially in the context that India uses indigenously developed vaccines (Rotavac and Rotasiil) which is different from the rest of the world [71]. Whole genome characterization also helped detect the vaccine reassortant strains. Detection of novel genotypes like E6-NSP4, T6-NSP3 and H3-NSP5, unusual strains like OP-354, DS-1 like G1P[8], equine like strains is a constant reminder to continue genome level surveillance of RV strains for tracking virus evolution in India especially in the post vaccine era.

## Materials and methods

### Ethics statement and sample collection

This study was approved by the Institutional Review Board of Christian Medical College (CMC), Vellore. In this study samples were selected from the biorepository of stool samples already collected and stored for different community- and hospital-based rotavirus studies at the Wellcome Trust Research Laboratory, at CMC, Vellore. The rotavirus positive stool samples were obtained from our laboratory biorepository. These diarrheal samples were collected from children under 5 years of age as a part of rotavirus surveillance studies conducted both in the hospital or community settings. The stool samples were first screened for rotavirus particles by ELISA and the rotavirus positive samples were genotyped by RT-PCR or sanger sequencing.

### Selection of samples for whole genome sequencing

The VP7 and VP4 genotype and Sanger sequence data from the laboratory database were used to select a subset of stool samples for next generation sequencing (NGS). At least 3 strains each of G1P[8], G2P[4] and G12 genotypes were randomly selected from each year from 2002 to 2017. A small number of strains of G4, G6, G8 and G9 and uncharacterised genotypes were also included. A total of 127 Wa-like (88 G1P[8], 16 G12P[6], 12 G12P[8], 1 G12P[4], 2 G4, 2 G1P[x], 4 G9P[8], 1 G12P[x] and G1 + G12P[8]) and 80 DS-1-like strains (67 G2P[4], 2 G2P[8], 2 G2P[X], 1 G2P[4] + P[8], 4 G9P[4], 1 G8P[14], 1G6P[X], 1G9P[X] and 1 GXP[14]) were sequenced by NGS.

### Viral dsRNA extraction for NGS

Viral nucleic acid was extracted from 20%(v/v) fecal extract using QIAmp 96 Virus QIAcube HT kit on the QIAcube HT automated platform (Qiagen, Germany) according to manufacturer’s instructions.

### Amplification of RV genome segments and NGS

#### Genotype confirmation

Complementary DNA (cDNA) synthesized by reverse transcription using Moloney murine reverse transcriptase enzyme (superscript II MMLV-RT, Invitrogen, United States) and random primers (Invitrogen, United States) were used as templates for VP7 and VP4 typing by a hemi-nested multiplex PCR using published primers [72, 73]. The untyped samples and unusual strains were sequenced by Sanger sequencing.

#### Amplification of 11 segments

The protocol followed in this study for amplification of RVA 11 genes segment was standardized and developed through a collaboration with the J. Craig Venter Institute, La Jolla, CA, USA. Briefly, a one-step reverse transcription-polymerase chain reaction (RT-PCR) was used to amplify 11 genes from extracted RNA in four multiplex reactions using Superscript IV One-step RT-PCR kit (Invitrogen, United States) and gene specific primers (see Table S1). The Superscript IV PCR reaction uses Superscript IV reverse transcriptase enzyme for cDNA synthesis and a super fidelity Taq polymerase enzyme from amplification of DNA in consecutive steps. The gene segments amplified were confirmed by running the amplified product on 1% agarose gel. The PCR products were purified using AMPure XP magnetic beads (Beckman Coulter, United States) according to manufacturer’s specifications.

#### Next generation sequencing

Sequencing libraries were prepared using Illumina Nextera DNA flex library preparation kit following manufacturer’s guidelines (Illumina, USA). The quality of libraries was assessed using Tapestation D1000 screen tape auto electrophoresis system (Agilent, USA) and Qubit fluorometric assay (Invitrogen, USA). Libraries were diluted to 12 picomoles and spiked with 5% PhiX sequencing control (Illumina, USA). NGS was carried out on an Illumina MiSeq sequencing platform (Illumina, USA) using MiSeq Reagent Kit v3 for 600 cycles with 300 bp paired end reads method.

### Whole genome sequence assembly

45 sample reads were assembled using CLC genomics Workbench (Qiagen) [74] and the remainder were assembled using Geneious R11/Prime software (Dotmatics, United States) [75]. Reads were first assembled using de-novo assembly followed by reference assembly to obtain full length sequences with a depth coverage greater than 100X. The length of open reading frames (ORFs) for gene segments were VP7 (981), VP4 (2328), VP1 (Wa-like 3283/DS-1-like 3267), VP2 (Wa-like 2685/ DS-1-like 2640), VP3 (2508), VP6 (1194), NSP1 (1461), NSP2 (954), NSP3 (Wa-like 933/DS-1-like 942), NSP4 (528), NSP5 (Wa-like 594/DS-1-like 603) base pairs.

### Accession numbers

The sequences for all the study strains were deposited to GenBank under accession numbers listed in the Supplementary File 10.

### Genotype assignment

Genotypes to each gene sequence was assigned using ViPR (Virus Pathogen Resource) Rotavirus A genotype determination tool (This web tool has now been upgraded as RIVM Rotavirus A genotyping tool version 1.0, manuscript is yet in preparation) [76]. After the genotype assignment, the strains were classified into genogroups Wa-like (genogroup 1) or DS-1-like (genogroup 2) for further analysis.

### Distance and phylogenetic analysis

#### Preparation of datasets

Fasta files of all the sequenced study strains were used for analysis. Pre-existing RV sequences were downloaded from GenBank database that included the primordial (referred as human classical rotavirus strains) RV strains from the globe (details in Table S4) Sequence similarity was analysed using p-distance method. Genetic distances were calculated for all the genes. Similarities were calculated within the study sequences (Table S5-S6) and in comparison, with several RVA reference sequences from GenBank (Table S7-S9). To compare Wa-like study sequences, human classical strains of genotypes G1P[8] (Wa, KU, AM06-1, Dhaka16), G12P[6] (Dhaka12, Matlab13, GER172), G12P[8] (B4633, GER126, Dhaka25), G3P[8] (P), G4P[8] (DC-2241), G9P[8] (WI61), and G4P[6] (ST3) were used. Vaccine strain sequences from 116E and Rotarix strain were also included for the analysis. To compare DS-1-like study sequences, human classical G2P[4] strain sequences (116E3D and DS-1), vaccine strain sequences (RotaTeq), DS-1 like G1P[8] (UFS-1971 and UFS-1973), classical Equine like strain sequences (IS1078, S13-30, S13-45), recent strains (BEN-7194 and BEN-7196) were included Likewise, Wa-like and DS-1-like human strains circulating in India (referred as Indian reference strains) were included in the distance analysis. For the phylogenetic analysis, apart from human classical rotavirus strain and Indian reference strains, few strains of animal origin were included from GenBank.

#### Bioinformatics pipeline

Sequence alignment was carried out in Aliview software using MUSCLE algorithm [77]. Nucleotide and deduced amino acid sequence identities among strains were calculated for each gene using *p*-distance algorithm in MEGA 7 software [78]. The Model Finder program was used to identify the optimal substitution model that best fit sequence datasets using Bayesian Information Criterion (BIC) [79]. Maximum likelihood trees were constructed using IQTree-2 software with 1000 bootstrap replicates [80, 81]. Maximum likelihood trees for each gene were built using the best substitution model according to the Bayesian criterion. The trees were visualized using iTOL online server and the sub-genotype clusters were defined based on branching patterns and bootstrap value [82]. Where sub-genotype cluster is defined as a distinct phylogenetic branch observed within a genotype containing the sequences sharing high similarity (> 95%) and supported by a bootstrap value of > 70%. Branches with bootstrap higher than 70 were considered for inference. The clusters with large number of leaves were collapsed for easy visualization. The human sub genotypic clusters of each genotype were read as HC-1 to HC-N, in the order of its appearance in the tree from top to bottom. The sequences that do not group with any reference other study strain sequences exist as a singlet leaf. For comparison, various classical strains, vaccine strains, reassortant wild-type human strains and wild-type animal strains were included (Refer Table S4).

### Electronic supplementary material

Below is the link to the electronic supplementary material.

Additional file 1 Table S1. List of primers for Rotavirus 11 genes amplification. Additional file 2: Table S2. Whole genome constellation of Wa-like strains sequenced from Vellore, arranged in the order of year of collection. Green colour box indicates the complete sequence of the classical gene, yellow colour box indicates the complete sequence of the reassortant genes, and the red colour box indicates the missing gene(the segment which could not be sequenced). Additional file 3: Table S3. Whole genome constellation of DS-1-like strains sequenced from Vellore, arranged in the order of year of collection. Green colour box indicates the complete sequence of the classical gene, yellow colour box indicates the complete sequence of the reassortant genes, and the red colour box indicates the missing gene(the segment which could not be sequenced). Additional file 4: Table S4. Details of the reference strains used for distance analysis and phylogenetic analysis. Additional file 5: Table S5. Observed sequence identities among the protein coding regions of the 11 RVA genes among different genotypes of the sequenced Wa-like strains. (NT-Nucleotide identity, AA-Amino Acid identity in percentage). Additional file 6: Table S6. Observed sequence identities among the protein coding regions of the 11 RVA genes among different genotypes of the sequenced DS-1-like strains. (NT-Nucleotide identity, AA-Amino Acid identity in percentage). Additional file 7: Table S7. Observed sequence identities of the protein coding sequences of Wa-like strains compared with reference strains from GenBank. The highest and the lowest identity in comparison with classical strains is highlighted in orange and green respectively. Identity score in comparison to circulating human strains from India are highlighted in red (NT-Nucleotide identity, AA-Amino Acid identity in percentage). Additional file 8: Table S8. Observed sequence identities of the protein coding sequences of VP4 and VP7 genes of Wa-like strains compared with reference strains from GenBank. The highest and the lowest identity in comparison with reference strains is highlighted in orange and green respectively. (NT-Nucleotide identity, AA-Amino Acid identity in percentage). Additional file 9: Table S9. Observed sequence identities of the protein coding sequences of DS-1-like strains compared with reference strains from GenBank. The highest and the lowest identity in comparison with classical strains is highlighted in orange and green respectively. Identity score in comparison to circulating human strains from India are highlighted in red (NT-Nucleotide identity, AA-Amino Acid identity in percentage).

Supplementary material 10: GenBank Accession Numbers of 11 segments of 207 study strains sequenced in the study.

## Data Availability

The genome sequences of all study strains are publicly available in GenBank database (https://www.ncbi.nlm.nih.gov/nuccore) for which the accession number are listed in Supplementary file 10.

## List of Abbreviations

BP: Base Pairs
RV: Rotavirus
RVA: Rotavirus A
NT: Nucleotide
AA: Amino Acid
VP: Viral Protein
NSP: Non-Structural Protein
cDNA: Complementary Deoxyribonucleotide Nucleic Acid
RNA: Ribonucleotide Nucleic Acid
MMLV-RT: Moloney Murine Leukaemia Virus - Reverse Transcriptase
PCR: Polymerase Chain Reaction
RT-PCR: Reverse Transcriptase- Polymerase Chain Reaction
HC: Human Cluster
GG: Genogroup

## References

[1] Morbidity N, GBD Diarrhoeal Diseases Collaborators Estimates of Global, Regional, and (2017). Mortality, and Aetiologies of Diarrhoeal Diseases: a systematic analysis for the global burden of Disease Study 2015. Lancet Infect Dis.

[2] Morbidity N, Estimates of the Global, Regional, and (2018). Mortality, and Aetiologies of Diarrhoea in 195 countries: a systematic analysis for the global burden of Disease Study 2016. Lancet Infect Dis.

[3] Cohen AL, Platts-Mills JA, Nakamura T, Operario DJ, Antoni S, Mwenda JM, Weldegebriel G, Rey-Benito G, Oliveira LH, Ortiz C (2022). Aetiology and incidence of Diarrhoea requiring hospitalisation in children under 5 years of age in 28 low-income and Middle-Income Countries: findings from the Global Pediatric Diarrhea Surveillance Network. BMJ Global Health.

[4] Greenberg HB, Estes MK, Rotaviruses (2009). From pathogenesis to vaccination. Gastroenterology.

[5] Estes MK, Cohen J (1989). Rotavirus Gene structure and function. Microbiol Rev.

[6] Matthijnssens J, Ciarlet M, Rahman M, Attoui H, Bányai K, Estes MK, Gentsch JR, Iturriza-Gómara M, Kirkwood CD, Martella V (2008). Recommendations for the classification of Group A Rotaviruses using all 11 genomic RNA segments. Arch Virol.

[7] Matthijnssens J, Ciarlet M, Heiman E, Arijs I, Delbeke T, McDonald SM, Palombo EA, Iturriza-Gómara M, Maes P, Patton JT (2008). Full genome-based classification of Rotaviruses reveals a common origin between Human Wa-Like and Porcine Rotavirus strains and Human DS-1-like and bovine Rotavirus strains. J Virol.

[8] Rotavirus Classification Working Group.: RCWG Available online: https://rega.kuleuven.be/cev/viralmetagenomics/virus-classification/rcwg (accessed on 10 September 2022).

[9] Ramig RF (1997). Genetics of the Rotaviruses. Annu Rev Microbiol.

[10] Hoxie I, Dennehy JJ (2020). Intragenic recombination influences Rotavirus Diversity and Evolution. Virus Evol.

[11] Phan TG, Okitsu S, Maneekarn N, Ushijima H (2007). Evidence of Intragenic recombination in G1 Rotavirus VP7 genes. J Virol.

[12] Jere KC, Mlera L, Page NA, van Dijk AA, O’Neill HG (2011). Whole genome analysis of multiple rotavirus strains from a single stool specimen using sequence-independent amplification and 454® pyrosequencing reveals evidence of Intergenotype Genome Segment recombination. Infect Genet Evol.

[13] Suzuki Y, Gojobori T, Nakagomi O (1998). Intragenic recombinations in Rotaviruses. FEBS Lett.

[14] Ghosh S, Kobayashi N (2011). Whole-genomic analysis of Rotavirus strains: current status and future prospects. Future Microbiol.

[15] Jere KC, Chaguza C, Bar-Zeev N, Lowe J, Peno C, Kumwenda B, Nakagomi O, Tate JE, Parashar UD, Heyderman RS (2018). Emergence of double- and triple-gene reassortant G1P[8] Rotaviruses possessing a DS-1-Like Backbone after Rotavirus Vaccine introduction in Malawi. J Virol.

[16] Maringa WM, Simwaka J, Mwangi PN, Mpabalwani EM, Mwenda JM, Mphahlele MJ, Seheri ML, Nyaga MM (2021). Whole genome analysis of human Rotaviruses reveals single gene reassortant rotavirus strains in Zambia. Viruses.

[17] Nakagomi T, Nguyen MQ, Gauchan P, Agbemabiese CA, Kaneko M, Do LP, Vu TD, Nakagomi O (2017). Evolution of DS-1-like G1P[8] double-gene reassortant rotavirus A strains causing gastroenteritis in children in Vietnam in 2012/2013. Arch Virol.

[18] Komoto S, Tacharoenmuang R, Guntapong R, Ide T, Tsuji T, Yoshikawa T, Tharmaphornpilas P, Sangkitporn S, Taniguchi K (2016). Reassortment of Human and Animal Rotavirus Gene Segments in emerging DS-1-Like G1P[8] Rotavirus strains. PLoS ONE.

[19] Iturriza-Gómara M, Isherwood B, Desselberger U, Gray J (2001). Reassortment in vivo: driving force for diversity of human rotavirus strains isolated in the United Kingdom between 1995 and 1999. J Virol.

[20] Varghese T, Alokit Khakha S, Giri S, Nair NP, Badur M, Gathwala G, Chaudhury S, Kaushik S, Dash M, Mohakud NK (2021). Rotavirus strain distribution before and after introducing Rotavirus Vaccine in India. Pathogens.

[21] Babji S, Arumugam R, Priyahemavathy R, Sriraman A, Sarvanabhavan A, Manickavasagam P, Simon A, Aggarwal I, Moses PD, Arora R (2018). Genotype distribution of Group A Rotavirus from Southern India, 2005–2016. Vaccine.

[22] Zeller M, Heylen E, Damanka S, Pietsch C, Donato C, Tamura T, Kulkarni R, Arora R, Cunliffe N, Maunula L (2015). Emerging OP354-Like P[8] Rotaviruses have rapidly dispersed from Asia to other continents. Mol Biol Evol.

[23] Libonati MH, Dennis AF, Ramani S, McDonald SM, Akopov A, Kirkness EF, Kang G, Patton JT (2014). Absence of genetic differences among G10P[11] Rotaviruses Associated with asymptomatic and symptomatic neonatal infections in Vellore, India. J Virol.

[24] Giri S, Kumar CPG, Khakha SA, Chawla-Sarkar M, Gopalkrishna V, Chitambar SD, Ray P, Venkatasubramanian S, Borkakoty BJ, Roy S (2020). Diversity of Rotavirus genotypes circulating in children < 5 years of Age hospitalized for Acute Gastroenteritis in India from 2005 to 2016: analysis of temporal and Regional genotype variation. BMC Infect Dis.

[25] Mishra V, Awasthi S, Nag VL, Tandon R (2010). Genomic diversity of Group A Rotavirus strains in patients aged 1–36 months admitted for Acute Watery Diarrhoea in Northern India: A Hospital-Based study. Clin Microbiol Infect.

[26] Reesu R, Bhattacharya D, Chaaithanya IK, Muruganandam N, Bharadwaj AP, Singhania M, Sugunan AP, Vijayachari P. Emergence of an Unusual Genotype of Rotavirus in Andaman and Nicobar Islands, India. *Intervirology* 2013, *56*, 134–139, 10.1159/000342219. 10.1159/000342219 23295640

[27] Mathew MA, Paulose A, Chitralekha S, Nair MKC, Kang G, Kilgore P (2014). Prevalence of Rotavirus Diarrhea among Hospitalized under-five children. Indian Pediatr.

[28] Tiku VR, Sharma S, Verma A, Kumar P, Raghavendhar S, Aneja S, Paul VK, Bhan MK, Ray P (2014). Rotavirus Diversity among Diarrheal Children in Delhi, India during 2007–2012. Vaccine.

[29] Mukherjee A, Chattopadhyay S, Bagchi P, Dutta D, Singh NB, Arora R, Parashar UD, Gentsch JR, Chawla-Sarkar M (2010). Surveillance and molecular characterization of Rotavirus strains circulating in Manipur, North-Eastern India: increasing prevalence of emerging G12 strains. Infect Genet Evol.

[30] Chakravarti A, Chauhan MS, Sharma A, Verma V (2010). Distribution of human rotavirus G and P genotypes in a hospital setting from Northern India. Southeast Asian J Trop Med Public Health.

[31] Saluja T, Sharma SD, Gupta M, Kundu R, Kar S, Dutta A, Silveira M, Singh JV, Kamath VG, Chaudhary A et al. A Multicenter Prospective Hospital-Based Surveillance to Estimate the Burden of Rotavirus Gastroenteritis in Children Less than Five Years of Age in India. *Vaccine* 2014, *32 Suppl 1*, A13-19, 10.1016/j.vaccine.2014.03.030. 10.1016/j.vaccine.2014.03.030 25091667

[32] Das S, Varghese V, Chaudhury S, Barman P, Mahapatra S, Kojima K, Bhattacharya SK, Krishnan T, Ratho RK, Chhotray GP (2003). Emergence of Novel Human Group A Rotavirus G12 strains in India. J Clin Microbiol.

[33] Santos N, Hoshino Y (2005). Global distribution of Rotavirus Serotypes/Genotypes and its implication for the development and implementation of an effective Rotavirus Vaccine. Rev Med Virol.

[34] Ide T, Higo-Moriguchi K, Komoto S, Htun KW, Myint YY, Myat TW, Thant KZ, Thu HM, Win MM, Oo HN (2016). High prevalence of G12 Human Rotaviruses in children with gastroenteritis in Myanmar. Jpn J Infect Dis.

[35] Pun SB, Nakagomi T, Sherchand JB, Pandey BD, Cuevas LE, Cunliffe NA, Hart CA, Nakagomi O (2007). Detection of G12 Human Rotaviruses in Nepal. Emerg Infect Dis.

[36] Rahman M, Sultana R, Ahmed G, Nahar S, Hassan ZM, Saiada F, Podder G, Faruque ASG, Siddique AK, Sack DA (2007). Prevalence of G2P[4] and G12P[6] Rotavirus, Bangladesh. Emerg Infect Dis.

[37] Rahman M, Matthijnssens J, Yang X, Delbeke T, Arijs I, Taniguchi K, Iturriza-Gómara M, Iftekharuddin N, Azim T, Van Ranst M (2007). Evolutionary history and global spread of the Emerging G12 Human Rotaviruses. J Virol.

[38] Roy S, Esona MD, Kirkness EF, Akopov A, McAllen JK, Wikswo ME, Cortese MM, Payne DC, Parashar UD, Gentsch JR (2014). Comparative genomic analysis of Genogroup 1 (Wa-like) Rotaviruses circulating in the USA, 2006–2009. Infect Genet Evol.

[39] Esona MD, Gautam R, Katz E, Jaime J, Ward ML, Wikswo ME, Betrapally NS, Rustempasic SM, Selvarangan R, Harrison CJ (2021). Comparative genomic analysis of Genogroup 1 and genogroup 2 rotaviruses circulating in seven US Cities, 2014–2016. Virus Evol.

[40] McDonald SM, McKell AO, Rippinger CM, McAllen JK, Akopov A, Kirkness EF, Payne DC, Edwards KM, Chappell JD, Patton JT (2012). Diversity and Relationships of Cocirculating Modern Human Rotaviruses revealed using Large-Scale Comparative Genomics. J Virol.

[41] Agbla JM, Esona MD, Agbankpe AJ, Capo-Chichi A, Gautam R, Dougnon TV, Razack O, Bowen MD, Bankole HS (2020). Molecular characteristics of Rotavirus genotypes circulating in the South of Benin, 2016–2018. BMC Res Notes.

[42] Dennis AF, McDonald SM, Payne DC, Mijatovic-Rustempasic S, Esona MD, Edwards KM, Chappell JD, Patton JT (2014). Molecular Epidemiology of Contemporary G2P[4] human Rotaviruses cocirculating in a single U.S. community: footprints of a globally transitioning genotype. J Virol.

[43] Fujii Y, Nakagomi T, Nishimura N, Noguchi A, Miura S, Ito H, Doan YH, Takahashi T, Ozaki T, Katayama K (2014). Spread and predominance in Japan of Novel G1P[8] double-reassortant rotavirus strains possessing a DS-1-like genotype Constellation typical of G2P[4] strains. Infect Genet Evol.

[44] Yamamoto SP, Kaida A, Kubo H, Iritani N (2014). Gastroenteritis outbreaks caused by a DS-1-like G1P[8] Rotavirus strain, Japan, 2012–2013. Emerg Infect Dis.

[45] Doan YH, Suzuki Y, Fujii Y, Haga K, Fujimoto A, Takai-Todaka R, Someya Y, Nayak MK, Mukherjee A, Imamura D (2017). Complex reassortment events of unusual G9P[4] Rotavirus strains in India between 2011 and 2013. Infect Genet Evol.

[46] Pradhan GN, Walimbe AM, Chitambar SD (2016). Molecular characterization of emerging G9P[4] Rotavirus strains possessing a rare E6 NSP4 or T1 NSP3 genotype on a Genogroup-2 backbone using a Refined classification Framework. J Gen Virol.

[47] Quaye O, McDonald S, Esona MD, Lyde FC, Mijatovic-Rustempasic S, Roy S, Banegas DJC, Quiñonez YM, Chinchilla BL, Santiago FG (2013). Rotavirus G9P[4] in 3 countries in Latin America, 2009–2010. Emerg Infect Dis.

[48] Sharma S, Paul VK, Bhan MK, Ray P (2009). Genomic characterization of Nontypeable Rotaviruses and detection of a rare G8 strain in Delhi, India. J Clin Microbiol.

[49] Pradhan GN, Chitambar SD (2018). Genetic analysis of Rotavirus G2P[4] strains in Pune, Western India: circulation of a Novel Reassortant Bearing E6 NSP4 genotype. Arch Virol.

[50] da Silva MFM, Rose TL, Gómez MM, Carvalho-Costa FA, Fialho AM, de Assis RMS, de Andrade J, da Volotão SR, de Leite E (2015). G. G1P[8] species a Rotavirus over 27 years–pre- and post-vaccination eras–in Brazil: full genomic Constellation Analysis and no evidence for selection pressure by Rotarix® Vaccine. Infect Genet Evol.

[51] Rose TL, Marques da Silva MF, Goméz MM, Resque HR, Ichihara MYT, Volotão E, de Leite M (2013). Evidence of vaccine-related reassortment of Rotavirus, Brazil, 2008–2010. Emerg Infect Dis.

[52] Boom JA, Sahni LC, Payne DC, Gautam R, Lyde F, Mijatovic-Rustempasic S, Bowen MD, Tate JE, Rench MA, Gentsch JR (2012). Symptomatic infection and detection of vaccine and vaccine-reassortant rotavirus strains in 5 children: a Case Series. J Infect Dis.

[53] Bucardo F, Rippinger CM, Svensson L, Patton JT, Vaccine-Derived NSP (2012). 2 segment in Rotaviruses from Vaccinated Children with Gastroenteritis in Nicaragua. Infect Genet Evol.

[54] Glass RI, Bhan MK, Ray P, Bahl R, Parashar UD, Greenberg H, Rao CD, Bhandari N, Maldonado Y, Ward RL (2005). Development of candidate Rotavirus Vaccines derived from neonatal strains in India. J Infect Dis.

[55] Cook N, Bridger J, Kendall K, Gomara MI, El-Attar L, Gray J (2004). The zoonotic potential of Rotavirus. J Infect.

[56] Dóró R, Farkas SL, Martella V, Bányai K (2015). Zoonotic transmission of Rotavirus: Surveillance and Control. Expert Rev Anti-infective Therapy.

[57] Simsek C, Corman VM, Everling HU, Lukashev AN, Rasche A, Maganga GD, Binger T, Jansen D, Beller L, Deboutte W (2021). At least seven distinct Rotavirus genotype constellations in bats with evidence of reassortment and zoonotic transmissions. mBio.

[58] Matthijnssens J, De Grazia S, Piessens J, Heylen E, Zeller M, Giammanco GM, Bányai K, Buonavoglia C, Ciarlet M, Martella V (2011). Multiple reassortment and Interspecies transmission events contribute to the diversity of Feline, Canine and Feline/Canine-like Human Group A Rotavirus strains. Infect Genet Evol.

[59] Matthijnssens J, Potgieter CA, Ciarlet M, Parreño V, Martella V, Bányai K, Garaicoechea L, Palombo EA, Novo L, Zeller M (2009). Are human P[14] Rotavirus strains the result of Interspecies Transmissions from Sheep or other ungulates that belong to the mammalian order Artiodactyla?. J Virol.

[60] Ghosh S, Kobayashi N (2014). Exotic rotaviruses in animals and rotaviruses in exotic animals. Virusdisease.

[61] Zeller M, Donato C, Trovão NS, Cowley D, Heylen E, Donker NC, McAllen JK, Akopov A, Kirkness EF, Lemey P (2015). Genome-wide evolutionary analyses of G1P[8] strains isolated before and after Rotavirus Vaccine introduction. Genome Biol Evol.

[62] Matthijnssens J, Heylen E, Zeller M, Rahman M, Lemey P, Van Ranst M (2010). Phylodynamic analyses of Rotavirus genotypes G9 and G12 underscore their potential for Swift Global Spread. Mol Biol Evol.

[63] Nagaoka Y, Tatsumi M, Tsugawa T, Yoto Y, Tsutsumi H (2012). Phylogenetic and computational structural analysis of VP7 gene of Group a human Rotavirus G1P[8] strains obtained in Sapporo, Japan from 1987 to 2000. J Med Virol.

[64] Jenkins GM, Rambaut A, Pybus OG, Holmes EC (2002). Rates of Molecular Evolution in RNA viruses: a quantitative phylogenetic analysis. J Mol Evol.

[65] Donker NC, Kirkwood CD (2012). Selection and evolutionary analysis in the nonstructural protein NSP2 of Rotavirus A. Infect Genet Evol.

[66] Nguyen TA, Hoang LP, Pham LD, Hoang KT, Okitsu S, Mizuguchi M, Ushijima H (2008). Use of sequence analysis of the VP4 gene to Classify recent vietnamese Rotavirus isolates. Clin Microbiol Infect.

[67] Dong H-J, Qian Y, Zhang Y, Deng L, Zhao L-Q, Zhu R-N, Chen D-M, Liu L-Y, Jia L-P (2011). [Investigation of a novel VP4 subgenotype of rotavirus in children with diarrhea in Beijing during 2009–2010]. Bing Du Xue Bao.

[68] Komoto S, Ide T, Negoro M, Tanaka T, Asada K, Umemoto M, Kuroki H, Ito H, Tanaka S, Ito M (2018). Characterization of unusual DS-1-like G3P[8] Rotavirus strains in children with Diarrhea in Japan. J Med Virol.

[69] Dóró R, Marton S, Bartókné AH, Lengyel G, Agócs Z, Jakab F, Bányai K (2016). Equine-like G3 Rotavirus in Hungary, 2015 - is it a Novel Intergenogroup Reassortant Pandemic strain?. Acta Microbiol Immunol Hung.

[70] Malasao R, Saito M, Suzuki A, Imagawa T, Nukiwa-Soma N, Tohma K, Liu X, Okamoto M, Chaimongkol N, Dapat C (2015). Human G3P[4] Rotavirus obtained in Japan, 2013, possibly emerged through a human-equine Rotavirus Reassortment Event. Virus Genes.

[71] Bergman H, Henschke N, Hungerford D, Pitan F, Ndwandwe D, Cunliffe N, Soares-Weiser K (2021). Vaccines for preventing Rotavirus Diarrhoea: vaccines in Use. Cochrane Database Syst Rev.

[72] Kang G, Desai R, Arora R, Chitamabar S, Naik TN, Krishnan T, Deshpande J, Gupte MD, Venkatasubramaniam S, Gentsch JR (2013). Diversity of circulating Rotavirus strains in children hospitalized with Diarrhea in India, 2005–2009. Vaccine.

[73] Iturriza-Gomara M, Green J, Brown DW, Desselberger U, Gray JJ (1999). Comparison of specific and Random Priming in the reverse transcriptase polymerase chain reaction for genotyping Group A Rotaviruses. J Virol Methods.

[74] Home - QIAGEN Digital Insights Available online.: https://digitalinsights.qiagen.com/ (accessed on 21 September 2022).

[75] Kearse M, Moir R, Wilson A, Stones-Havas S, Cheung M, Sturrock S, Buxton S, Cooper A, Markowitz S, Duran C (2012). Geneious Basic: an Integrated and Extendable Desktop Software platform for the Organization and analysis of sequence data. Bioinformatics.

[76] Virus Pathogen Database and Analysis Resource (ViPR). - Reoviridae - Rotavirus A Genotype Determination Available online: https://www.viprbrc.org/brc/rvaGenotyper.spg?method=ShowCleanInputPage&decorator=reo (accessed on 11 September 2022).

[77] Larsson A, AliView (2014). A fast and lightweight alignment viewer and editor for large datasets. Bioinformatics.

[78] Kumar S, Stecher G, Tamura K (2016). MEGA7: Molecular Evolutionary Genetics Analysis Version 7.0 for bigger datasets. Mol Biol Evol.

[79] Kalyaanamoorthy S, Minh BQ, Wong TKF, von Haeseler A, Jermiin LS, ModelFinder (2017). Fast model selection for accurate phylogenetic estimates. Nat Methods.

[80] Minh BQ, Schmidt HA, Chernomor O, Schrempf D, Woodhams MD, von Haeseler A, Lanfear R (2020). IQ-TREE 2: New Models and efficient methods for phylogenetic inference in the genomic era. Mol Biol Evol.

[81] Hoang DT, Chernomor O, von Haeseler A, Minh BQ, Vinh LS (2018). UFBoot2: improving the Ultrafast bootstrap approximation. Mol Biol Evol.

[82] Letunic I, Bork P (2021). Interactive tree of life (ITOL) v5: an Online Tool for phylogenetic Tree Display and Annotation. Nucleic Acids Res.

