# Supplementary material for: Whole-genome characterization of common rotavirus strains circulating in Vellore, India from 2002 to 2017: emergence of non-classical genomic constellations
Source: Gut Pathog. 2023 Sep 20;15:44. doi: 10.1186/s13099-023-00569-6 (PMC10510252; doi:10.1186/s13099-023-00569-6)
Supplement: Supplementary file 1 — Additional file 1 Table S1. List of primers for Rotavirus 11 genes amplification. Additional file 2: Table S2. Whole genome constellation of Wa-like strains sequenced from Vellore, arranged in the order of year of collection. Green colour box indicates the complete sequence of the classical gene, yellow colour box indicates the complete sequence of the reassortant genes, and the red colour box indicates the missing gene(the segment which could not be sequenced). Additional file 3: Table S3. Whole genome constellation of DS-1-like strains sequenced from Vellore, arranged in the order of year of collection. Green colour box indicates the complete sequence of the classical gene, yellow colour box indicates the complete sequence of the reassortant genes, and the red colour box indicates the missing gene(the segment which could not be sequenced). Additional file 4: Table S4. Details of the reference strains used for distance analysis and phylogenetic analysis. Additional file 5: Table S5. Observed sequence identities among the protein coding regions of the 11 RVA genes among different genotypes of the sequenced Wa-like strains. (NT-Nucleotide identity, AA-Amino Acid identity in percentage). Additional file 6: Table S6. Observed sequence identities among the protein coding regions of the 11 RVA genes among different genotypes of the sequenced DS-1-like strains. (NT-Nucleotide identity, AA-Amino Acid identity in percentage). Additional file 7: Table S7. Observed sequence identities of the protein coding sequences of Wa-like strains compared with reference strains from GenBank. The highest and the lowest identity in comparison with classical strains is highlighted in orange and green respectively. Identity score in comparison to circulating human strains from India are highlighted in red (NT-Nucleotide identity, AA-Amino Acid identity in percentage). Additional file 8: Table S8. Observed sequence identities of the protein coding sequences of VP4 and VP7 genes of Wa-like str [file 13099_2023_569_MOESM1_ESM.docx]

| **Gene** | **Segment** | **Primer name** | **Sequence 5’-3’** | **Amplicon length (BP)** | **Reference** |
| --- | --- | --- | --- | --- | --- |
| **VP1** | **1** | **VP1_NGS_F** | TGTAAAACGACGGCCAGTGGCTATTAAAGCTGTAC | **3302** | [1] |
|  |  | **VP1_NGS_R** | CAGGAAACAGCTATGACCGGTCACATCTAAGCAC |  |  |
| **VP2** | **2** | **VP2_NGS_F** | GGCTATTAAAGGCTCAATG | **2723** | JCVI |
|  |  | **VP2_NGS_R** | GGTCATATCTCCACAGTG |  |  |
| **VP3** | **3** | **VP3A_NGS_F** | GTTTTACCTCTGATGGTG | **2568** | JCVI |
|  |  | **VP3A_NGS_R** | GGTCACATCATGACTAG |  |  |
|  |  | **VP3E_NGS_F** | GGCTWTTAAAGCARTATTAGTAGTG | **2505** | [2] |
|  |  | **VP3E_NGS_R** | TGACYAGTGTGTTAAGTTYTAGC |  |  |
|  |  | **VP3F_NGS_F** | GCCGGAGCTCTGCAGATATCGGCTWTTAAAGC | **2565** | [3] |
|  |  | **VP3F_NGS_R** | GCCGGAGCTCTGCAGATATCGGTCACATCATG |  |  |
| **VP4** | **4** | **VP4_NGS_F** | TGTAAAACGACGGCCAGTGGCTATAAAATGGCTTC | **2362** | [1] |
|  |  | **VP4_NGS_R** | CAGGAAACAGCTATGACCGGTCACATCCTCAATAG |  |  |
| **VP6** | **6** | **VP6_NGS_F** | GGCTTTWAAACGAAGTCTTC | **1364** | JCVI |
|  |  | **VP6_NGS_R** | GGTCACATCCTCTCAC |  |  |
| **VP7** | **9** | **VP7_NGS_F** | GGCTTTAAAAGAGAGAATTTC | **1063** | JCVI |
|  |  | **VP7_NGS_R** | GGTCACATCRWACAATTC |  |  |
| **NSP1** | **5** | **NSP1_NGS_F** | TTTGAAAAGTCTTGTGGAAG | **1557** | JCVI |
|  |  | **NSP1_NGS_R** | GGTCACATTTTATGCTGC |  |  |
| **NSP2** | **8** | **NSP2_NGS_F** | CGTCTCAGTCGCCGTTTG | **1050** | JCVI |
|  |  | **NSP2_NGS_R** | AGCGCTTTCTATTCTTRC |  |  |
| **NSP3** | **7** | **NSP3_NGS_F** | GGCWTTTAATGCTTTTCAG | **1055** | JCVI |
|  |  | **NSP3_NGS_R** | CCATTTAGGTTTTTGACAG |  |  |
| **NSP4** | **10** | **NSP4_NGS_F** | GTTCTGTTCCGAGAGAGC | **748** | JCVI |
|  |  | **NSP4_NGS_R** | TCACRYTAAGACCRTTCC |  |  |
| **NSP5** | **11** | **NSP5_NGS_F** | TGTAAAACGACGGCCAGTGGCTTTWAAAGCGCTAC | **666** | [1] |
|  |  | **NSP5_NGS_R** | CAGGAAACAGCTATGACCGGTCACAAAACGGGAG |  |  |

Additional file 1:

Table S1: List of primers for Rotavirus 11 genes amplification

*JCVI- Primers designed by J. Craig Venter Institute, California, USA

1. Nyaga, M.M.; Stucker, K.M.; Esona, M.D.; Jere, K.C.; Mwinyi, B.; Shonhai, A.; Tsolenyanu, E.; Mulindwa, A.; Chibumbya, J.N.; Adolfine, H.; et al. Whole-Genome Analyses of DS-1-like Human G2P[4] and G8P[4] Rotavirus Strains from Eastern, Western and Southern Africa. *Virus Genes* **2014**, *49*, 196–207, doi:10.1007/s11262-014-1091-7.

2. Zeller, M.; Nuyts, V.; Heylen, E.; De Coster, S.; Conceição-Neto, N.; Van Ranst, M.; Matthijnssens, J. Emergence of Human G2P[4] Rotaviruses Containing Animal Derived Gene Segments in the Post-Vaccine Era. *Sci Rep* **2016**, *6*, 36841, doi:10.1038/srep36841.

3. Dung, T.T.N.; Duy, P.T.; Sessions, O.M.; Sangumathi, U.K.; Phat, V.V.; Tam, P.T.T.; To, N.T.N.; Phuc, T.M.; Hong Chau, T.T.; Chau, N.N.M.; et al. A Universal Genome Sequencing Method for Rotavirus A from Human Fecal Samples Which Identifies Segment Reassortment and Multi-Genotype Mixed Infection. *BMC Genomics* **2017**, *18*, 324, doi:10.1186/s12864-017-3714-6.

Additional file 2:

Table S2: Whole genome constellation of Wa-like strains sequenced from Vellore, arranged in the order of year of collection. Green colour box indicates the complete sequence of the classical gene, yellow colour box indicates the complete sequence of the reassortant genes, and the red colour box indicates the missing gene(the segment which could not be sequenced).

|  |  |  |  | WHOLE GENOME CONSTELLATION | | | | | | | | | | |
| --- | --- | --- | --- | --- | --- | --- | --- | --- | --- | --- | --- | --- | --- | --- |
| **SL.NO** | **Strain** | **Year** | **STRAIN ID** | **VP7** | **VP4** | **VP6** | **VP1** | **VP2** | **VP3** | **NSP1** | **NSP2** | **NSP3** | **NSP4** | **NSP5/6** |
| 1 | G1P[8] | 2002 | RVA/Human-wt/IND/RV-19/2002/G1P[8] | G1 | P[8] | I1 | R1 | C1 | M1 | A1 | N1 | T1 | E1 | H1 |
| 2 |  |  | RVA/Human-wt/IND/CRI-2157/2002/G1P[8] | G1 | P[8] | I1 | R1 | C1 | M1 | A1 | N1 | T1 | E1 | H1 |
| 3 |  |  | RVA/Human-wt/IND/CRI-2755/2002/G1P[8] | G1 | P[8] | I1 | R1 | C1 | M1 | A1 | N1 | T1 | E1 | H1 |
| 4 |  | 2003 | RVA/Human-wt/IND/RV-202/2003/G1P[8] | G1 | P[8] | I1 | R1 | C1 | M1 | A1 | N1 | T1 | E1 | H1 |
| 5 |  |  | RVA/Human-wt/IND/RV-219/2003/G1P[8] | G1 | P[8] | I1 | R1 | C1 | M1 | A1 | N1 | T1 | E1 | H1 |
| 6 |  |  | RVA/Human-wt/IND/CRI-3173/2003/G1P[8] | G1 | P[8] | I1 | R1 | C1 | M1 | A1 | N1 | T1 | E1 | H1 |
| 7 |  |  | RVA/Human-wt/IND/CRI-11702/2003/G1P[8] | G1 | P[8] | I1 | R1 | C1 | M1 | A1 | N1 | T1 | E1 | H1 |
| 8 |  |  | RVA/Human-wt/IND/CRI-13621/2003/G1P[8] | G1 | P[8] | I1 | R1 | C1 | M1 | A1 | N1 | T1 | E1 | H1 |
| 9 |  | 2004 | RVA/Human-wt/IND/RV-371/2004/G1P[8] | G1 | P[8] | I1 | R1 | C1 | M1 | A1 | N1 | T1 | E1 | H1 |
| 10 |  |  | RVA/Human-wt/IND/CRI-23589/2004/G1P[8] | G1 | P[8] | I1 | R1 | C1 | M1 | A1 | N1 | T1 | E1 | H1 |
| 11 |  |  | RVA/Human-wt/IND/CRI-23682/2004/G1P[8] | G1 | P[8] | I1 | R1 | C1 | M1 | A1 | N1 | T1 | E1 | H1 |
| 12 |  |  | RVA/Human-wt/IND/CRI-25360/2004/G1P[8] | G1 | P[8] | I1 | R1 | C1 | M1 | A1 | N1 | T1 | E1,E2 | H1 |
| 13 |  | 2005 | RVA/Human-wt/IND/CRI-36681/2005/G1P[8] | G1 | P[8] | I1 | R1 | C1 | M1 | A1 | N1 | T1 | E1 | H1 |
| 14 |  | 2006 | RVA/Human-wt/IND/C-107/2006/G1P[8] | G1 | P[8] | I1 | R1 | C1 | M1 | A1 | N1 | T1 | E1 | H1 |
| 15 |  |  | RVA/Human-wt/IND/C-320/2006/G1P[8] | G1 | P[8] | I1 | R1 | C1 | M1 | A1 | N1 | T1 | E1 | H1 |
| 16 |  |  | RVA/Human-wt/IND/C-0384/2006/G1P[8] | G1 | P[8] | I1 | R1 | C1 | M1 | A1 | N1 | T1 | E1 | H1 |
| 17 |  |  | RVA/Human-wt/IND/C-0385/2006/G1P[8] | G1 | P[8] | I1 | R1 | C1 | M1 | A1 | N1 | T1 | E1 | H1 |
| 18 |  | 2007 | RVA/Human-wt/IND/C-450/2007/G1P[8] | G1 | P[8] | I1 | R1 | C1 | M1 | A1 | N1 | T1 | E1 | H1 |
| 19 |  |  | RVA/Human-wt/IND/C-459/2007/G1P[8] | G1 | P[8] | I1 | R1 | C1 | M1 | A1 | N1 | T1 | E1 | H1 |
| 20 |  |  | RVA/Human-wt/IND/C-510/2007/G1P[8] | G1 | P[8] | I1 | R1 | C1 | M1 | A1 | N1 | T1 | E1 | H1 |
| 21 |  |  | RVA/Human-wt/IND/C-559/2007/G1P[8] | G1 | P[8] | I1 | R1 | C1 | M1 | A1 | N1 | T1 | E1 | H1 |
| 22 |  |  | RVA/Human-wt/IND/C-737/2007/G1P[8] | G1 | P[8] | I1 | R1 | C1 | M1 | A1 | N1 | T1 | E1 | H1 |
| 23 |  | 2008 | RVA/Human-wt/IND/C-744/2008/G1P[8] | G1 | P[8] | I1 | R1 | C1 | M1 | A1 | N1 | T1 | E1 | H1 |
| 24 |  |  | RVA/Human-wt/IND/C-793/2008/G1P[8] | G1 | P[8] | I1 | R1 | C1 | M1 | A1 | N1 | T1 | Ex | H1 |
| 25 |  |  | RVA/Human-wt/IND/C-826/2008/G1P[8] | G1 | P[8] | I1 | R1 | C1 | M1 | A1 | N1 | T1 | E1 | H1 |
| 26 |  |  | RVA/Human-wt/IND/C-929/2008/G1P[8] | G1 | P[8] | I1 | R1 | C1 | M1 | A1 | N1 | T1 | E1 | H1 |
| 27 |  |  | RVA/Human-wt/IND/C-0982/2008/G1P[8] | G1 | P[8] | I1 | R1 | C1 | M1 | A1 | N1 | T1 | E1 | H1 |
| 28 |  | 2009 | RVA/Human-wt/IND/C-1038/2009/G1P[8] | G1 | P[8] | I1 | R1 | C1 | M1 | A1 | N1 | T1 | E1 | H1 |
| 29 |  |  | RVA/Human-wt/IND/C-1089/2009/G1P[8] | G1 | P[8] | I1 | R1 | C1 | M1 | A1 | N1 | T1 | E1 | H1 |
| 30 |  |  | RVA/Human-wt/IND/C-1146/2009/G1P[8] | G1 | P[8] | I1 | R1 | C1 | M1 | A1 | N1 | T1 | E1 | H1 |
| 31 |  |  | RVA/Human-wt/IND/IRID-2631/2009/G1P[8] | G1 | P[8] | I2 | R1 | C1 | Mx | A1 | N1 | T1 | E1 | H1 |
| 32 |  |  | RVA/Human-wt/IND/LBF-054/2009/G1P[8] | G1 | P[8] | I1 | R1 | C1 | M1 | A1 | N1 | T1 | E1 | H1 |
| 33 |  |  | RVA/Human-wt/IND/IRID-4036/2009/G1P[8] | G1 | P[8] | I1 | R1 | C1 | Mx | A1 | N1 | T1 | E1 | H1 |
| 34 |  | 2010 | RVA/Human-wt/IND/IRID-4793/2010/G1P[8] | G1 | P[8] | I1 | R1 | C1 | M1 | A1 | N1 | T1 | E1 | H1 |
| 35 |  |  | RVA/Human-wt/IND/RO1-2802/2010/G1P[8] | G1 | P[8] | I1 | R1 | C1 | M1 | A1 | N1 | T1 | E1 | H1 |
| 36 |  |  | RVA/Human-wt/IND/C-1339/2010/G1P[8] | G1 | P[8] | I1 | R1 | C1 | M1 | A1 | N1 | T1 | E1 | H1 |
| 37 |  |  | RVA/Human-wt/IND/C-1383/2010/G1P[8] | G1 | P[8] | I1 | R1 | C1 | M1 | A1 | N1 | T1 | E1 | H1 |
| 38 |  |  | RVA/Human-wt/IND/RO1-9049/2010/G1P[8] | G1 | P[8] | I1 | R1 | C1 | M1 | A1 | N1 | T1 | E1 | H1 |
| 39 |  |  | RVA/Human-wt/IND/IN1000295_CMC_00001/2010/G1P[8] | G1 | P[8] | I1 | R1 | C1 | M1 | A1 | N1 | T1 | E1 | H1 |
| 40 |  |  | RVA/Human-wt/IND/IN1000324_CMC_00036/2010/G1P[8] | G1 | P[8] | I1 | R1 | C1 | M1 | A1 | N1 | T1 | E1 | H1 |
| 41 |  | 2011 | RVA/Human-wt/IND/RO1-16436/2011/G1P[8] | G1 | P[8] | I1 | R1 | C1 | M1 | A1 | N1 | T1 | E1 | H1 |
| 42 |  |  | RVA/Human-wt/IND/IN1002773_CMC_00010/2011/G1P[8] | G1 | P[8] | I1 | R1 | C1 | M1 | A1 | N1 | T1 | E1 | H1 |
| 43 |  |  | RVA/Human-wt/IND/LBF-4008/2011/G1P[8] | G1 | P[8] | I1 | R1 | C1 | M1 | A1 | N1 | T1 | E1 | H1 |
| 44 |  |  | RVA/Human-wt/IND/IN1002926_CMC_00011/2011/G1P[8] | G1 | P[8] | I1 | R1 | C1 | M1 | A1 | N1 | T1 | E1 | H1 |
| 45 |  |  | RVA/Human-wt/IND/IN1002928_CMC_00012/2011/G1P[8] | G1 | P[8] | I1 | R1 | C1 | M1 | A1 | N1 | T1 | E1 | H1 |
| 46 |  |  | RVA/Human-wt/IND/IN1002929_CMC_00013/2011/G1P[8] | G1 | P[8] | I1 | R1 | C1 | M1 | A1 | N1 | T1 | E1 | H1 |
| 47 |  |  | RVA/Human-wt/IND/LBF-4635/2011/G1P[8] | G1 | P[8] | I1 | R1 | C1 | Mx | A1 | N1 | T1 | E1 | H1 |
| 48 |  |  | RVA/Human-wt/IND/LBF-5453/2011/G1P[8] | G1 | P[8] | I1 | R1 | C1 | M1 | A1 | N1 | T1 | E1 | H1 |
| 49 |  |  | RVA/Human-wt/IND/IN1003708_CMC_00015/2011/G1P[8] | G1 | P[8] | I1 | R1 | C1 | M1 | A1 | N1 | T1 | E1 | H1 |
| 50 |  |  | RVA/Human-wt/IND/LBF-6279/2011/G1P[8] | G1 | P[8] | I1 | R1 | C1 | M1 | A1 | N1 | T1 | E1 | H1 |
| 51 |  | 2012 | RVA/Human-wt/IND/RVGE-169/2012/2012/G1P[8] | G1 | P[8] | I1 | R1 | C1 | M1 | A2 | N1 | T1,T2 | E1 | H1 |
| 52 |  |  | RVA/Human-wt/IND/IN1004468_CMC_00039/2012/G1P[8] | G1 | P[8] | I1 | R1 | C1 | M1 | A1 | N1 | T1 | E1 | H1 |
| 53 |  |  | RVA/Human-wt/IND/LBF-7652/2012/G1P[8] | G1 | P[8] | I1 | R1 | C1 | M1 | A1 | N1 | T1 | E1 | H1 |
| 54 |  |  | RVA/Human-wt/IND/IN1006236_CMC_00028/2012/G1P[8] | G1 | P[8] | I1 | R1 | C1 | M1 | A1 | N1 | T1 | E1 | H1 |
| 55 |  |  | RVA/Human-wt/IND/IN1006324_CMC_00029/2012/G1P[8] | G1 | P[8] | I1 | R1 | C1 | M1 | A1 | N1 | T1 | E1 | H1 |
| 56 |  |  | RVA/Human-wt/IND/IN1006379_CMC_00030/2012/G1P[8] | G1 | P[8] | I1 | R1 | C1 | M1 | A1 | N1 | T1 | E1 | H1 |
| 57 |  |  | RVA/Human-wt/IND/IN1006476_CMC_00031/2012/G1P[8] | G1 | P[8] | I1 | R1 | C1 | M1 | A1 | N1 | T1 | E1 | H1 |
| 58 |  |  | RVA/Human-wt/IND/IN1006722_CMC_00032/2012/G1P[8] | G1 | P[8] | I1 | R1 | C1 | M1 | A1 | N1 | T1 | E1 | H1 |
| 59 |  |  | RVA/Human-wt/IND/CM-0224/2012/2012/G1P[8] | G1 | P[8] | I1 | R1 | C1 | M1 | A1 | N1 | T1 | E1 | H1 |
| 60 |  |  | RVA/Human-wt/IND/IN1007249_CMC_00033/2012/G1P[8] | G1 | P[8] | I1 | R1 | C1 | M1 | A1 | N1 | T1 | E1 | H1 |
| 61 |  |  | RVA/Human-wt/IND/CM-0059/2012/G1P[8] | G1 | P[8] | I1 | R1 | C1 | M1 | A1 | N1 | T1 | E1 | H1 |
| 62 |  |  | RVA/Human-wt/IND/IN1008089_CMC_00045/2012/G1P[8] | G1 | P[8] | I1 | R1 | C1 | M1 | A1 | N1 | T1 | E1 | H1 |
| 63 |  | 2013 | RVA/Human-wt/IND/CM-0168/2013/G1P[8] | G1 | P[8] | I1 | R1 | C1 | M1 | A1 | N1 | T1 | E1 | H1 |
| 64 |  |  | RVA/Human-wt/IND/IN1008549_CMC_00034/2013/G1P[8] | G1 | P[8] | I1 | R1 | C1 | M1 | A1 | N1 | T1 | E1 | H1 |
| 65 |  |  | RVA/Human-wt/IND/IN1008580_CMC_00035/2013/G1P[8] | G1 | P[8] | I1 | R1 | C1 | M1 | A1 | N1 | T1 | E1 | H1 |
| 66 |  |  | RVA/Human-wt/IND/IN1008693_CMC_00047/2013/G1P[8] | G1 | P[8] | I1 | R1 | C1 | M1 | A1 | N1 | T1 | E1 | H1 |
| 67 |  |  | RVA/Human-wt/IND/CM-0373/2013/G1P[8] | G1 | P[8] | I1 | R1 | C1 | M1 | A1 | N1 | T1 | E1 | H1 |
| 68 |  |  | RVA/Human-wt/IND/CM-0547/2013/G1P[8] | G1 | P[8] | I1 | R1 | C1 | Mx | A1 | N2 | T1 | E1 | H1 |
| 69 |  | 2014 | RVA/Human-wt/IND/CM-0630/2014/G1P[8] | G1 | P[8] | I1 | R1 | C1 | M1 | A1 | N1 | T1 | E1 | H1 |
| 70 |  |  | RVA/Human-wt/IND/CM-0745/2014/G1P[8] | G1 | P[8] | I1 | R1 | C1 | M1 | A1 | N1 | T1 | E1 | H1 |
| 71 |  |  | RVA/Human-wt/IND/CM-0809/2014/G1P[8] | G1 | P[8] | I1 | R1 | C1 | M1 | A1 | N1 | T1 | E1 | H1 |
| 72 |  |  | RVA/Human-wt/IND/CM-0910/2014/G1P[8] | G1 | P[8] | I1 | R1 | C1 | M1 | A1 | N1 | T1 | E1 | H1 |
| 73 |  |  | RVA/Human-wt/IND/CM-0941/2014/G1P[8] | G1 | P[8] | I1 | R1 | C1 | M1 | A1 | N1 | T1 | E1 | H1 |
| 74 |  | 2015 | RVA/Human-wt/IND/CM-0987/2015/G1P[8] | G1 | P[8] | I1 | R1 | C1 | M1 | A1 | N1 | T1 | E1 | H1 |
| 75 |  |  | RVA/Human-wt/IND/CM-1050/2015/G1P[8] | G1 | P[8] | I1 | R1 | C1 | M1 | A1 | N1 | T1 | E1 | H1 |
| 76 |  |  | RVA/Human-wt/IND/CM-1243/2015/G1P[8] | G1 | P[8] | I1 | R1 | C1 | M1 | A1 | N1 | T1 | E1 | H1 |
| 77 |  |  | RVA/Human-wt/IND/CM-1340/2015/G1P[8] | G1 | P[8] | I1 | R1 | C1 | M1 | A1 | N1 | T1 | E1 | H1 |
| 78 |  | 2016 | RVA/Human-wt/IND/CM-1365/2016/G1P[8] | G1 | P[8] | I1 | R1 | C1 | M1 | A1 | N1 | T1 | E1 | H1 |
| 79 |  |  | RVA/Human-wt/IND/TN030002/2016/G1P[8] | G1 | P[8] | I1 | R1 | C1 | M1 | A1 | N1 | T1 | E1 | H1 |
| 80 |  |  | RVA/Human-wt/IND/CM-1455/2016/G1P[8] | G1 | P[8] | I1 | R1 | C1 | M1 | A1 | N1 | T1 | E1 | H1 |
| 81 |  |  | RVA/Human-wt/IND/CM-1463/2016/G1P[8] | G1 | P[8] | I1 | R1 | C1 | M1 | A1 | N1 | T1 | E1 | H1 |
| 82 |  |  | RVA/Human-wt/IND/TN020134/2016/G1P[8] | G1 | P[8] | I1 | R1 | C1 | M1 | A1 | N1 | T1 | E1 | H1 |
| 83 |  | 2017 | RVA/Human-wt/IND/TN010438/2017/G1P[8] | G1 | P[8] | I1 | R1 | C1 | M1 | A1 | N1 | T1 | E1 | H1 |
| 84 |  |  | RVA/Human-wt/IND/TN020175/2017/G1P[8] | G1 | P[8] | I1 | R1 | C1 | M1 | A1 | N1 | T1 | E1 | H1 |
| 85 |  |  | RVA/Human-wt/IND/TN010462/2017/G1P[8] | G1 | P[8] | I1 | R1 | C1 | M1 | A1 | N1 | T1 | E1 | H1 |
| 86 |  |  | RVA/Human-wt/IND/TN020197/2017/G1P[8] | G1 | P[8] | I1 | R1 | C1 | M1 | A1 | N1 | T1 | E1 | H1 |
| 87 |  |  | RVA/Human-wt/IND/TN020260/2017/G1P[8] | G1 | P[8] | I2 | R2 | C2 | Mx | A2 | N2 | T2 | E6 | H2 |
| 88 |  | NA | RVA/Human-wt/IND/PC/XXXX//G1P[8] | G1 | P[8] | I1 | R1 | C1 | M1 | A1 | N1 | T2 | E1 | H1 |
| 89 | G1P[x] | 2011 | RVA/Human-wt/IND/IN1002852_CMC_00037/2011/G1P[x] | G1 | P[X] | I1 | RX | CX | M1 | AX | NX | T1 | E1 | H1 |
| 90 |  | 2012 | RVA/Human-wt/IND/IN1004398_CMC_00021/2012/G1P[x] | G1 | P[X] | I1 | RX | CX | MX | A1 | N1 | T1 | E1 | H1 |

|  |  |  |  | WHOLE GENOME CONSTELLATION | | | | | | | | | | |
| --- | --- | --- | --- | --- | --- | --- | --- | --- | --- | --- | --- | --- | --- | --- |
| **SL.NO** |  |  | **STRAIN ID** | **VP7_9** | **VP4_4** | **VP6_6** | **VP1_1** | **VP2_2** | **VP3_3** | **NSP1_5** | **NSP2_8** | **NSP3_7** | **NSP4_10** | **NSP5/6_11** |
| 91 | G1+G12P[8] | 2007 | RVA/Human-wt/IND/C-0557/2007/G1+G12P[8] | G1+G12 | P[8] | I1 | R1 | C1 | M1 | A1,A2 | N1,N2 | T1,T2 | E1,E2 | H1 |
| 92 | G12P[4] | 2010 | RVA/Human-wt/IND/RO1-2748/2010/G12P[4] | G12 | P[4] | I1 | R1 | C1 | M1 | A1 | N1 | T1 | E1 | H1 |
| 93 | G12P[6] | 2005 | RVA/Human-wt/IND/CRI-32585/2005/G12P[6] | G12 | P[6] | I1 | R1 | C1 | M1 | A1 | N1 | T1 | E1 | H1 |
| 94 |  | 2009 | RVA/Human-wt/IND/RO1-2504/2009/G12P[6] | G12 | P[6] | I1 | R1 | C1 | M1 | A1 | N1 | T1 | E1 | H1 |
| 95 |  |  | RVA/Human-wt/IND/RO1-4649/2009/G12P[6] | G12 | P[6] | I1 | R1 | C1 | M1 | A1 | N1 | T1 | E1 | H1 |
| 96 |  | 2010 | RVA/Human-wt/IND/RO1-8625/2010/G12P[6] | G12 | P[6] | I1 | R1 | C1 | M1 | A1 | N1 | T1 | E1 | H1 |
| 97 |  |  | RVA/Human-wt/IND/RO1- 8980/2010/G12P[6] | G12 | P[6] | I1 | R2 | C1 | M1 | A1 | N1 | T1 | E1 | H1 |
| 98 |  |  | RVA/Human-wt/IND/RO1-14850/2010/G12P[6] | G12 | P[6] | I1 | R1 | C1 | M1 | A1 | N1 | T1 | E1 | H1 |
| 99 |  | 2011 | RVA/Human-wt/IND/RO1-24306/2011/G12P[6] | G12 | P[6] | I1 | R1 | C1 | M1 | A1 | N1 | T1 | E1 | H1 |
| 100 |  | 2012 | RVA/Human-wt/IND/RVGE-164/2012/G12P[6] | G12 | P[6] | I1 | R1 | C1 | M1 | A1 | N1 | T1 | E1 | H1 |
| 101 |  |  | RVA/Human-wt/IND/RVGE-505/2012/G12P[6] | G12 | P[6] | I1 | R1 | C1 | M1 | A1 | N1 | T1 | E1 | H1 |
| 102 |  | 2013 | RVA/Human-wt/IND/CM-0381/2013/G12P[6] | G12 | P[6] | I1 | R1 | C1 | M1 | A1 | N1 | T1 | E1 | H1 |
| 103 |  | 2014 | RVA/Human-wt/IND/CM-0625/2014/G12P[6] | G12 | P[6] | I1 | R1 | C1 | M1 | A1 | N1 | T1 | E1 | H1 |
| 104 |  |  | RVA/Human-wt/IND/CM-0684/2014/G12P[6] | G12 | P[6] | I2 | R1 | C1 | M1 | A1 | N1 | T1 | E1 | H1 |
| 105 |  |  | RVA/Human-wt/IND/CM-0694/2014/G12P[6] | G12 | P[6] | I1 | R1 | C1 | M1 | A1 | N1 | T1 | E1 | H1 |
| 106 |  |  | RVA/Human-wt/IND/CM-0713/2014/G12P[6] | G12 | P[6] | I1 | R1 | C1 | M1 | A1 | N1 | T1 | E1 | H1 |
| 107 |  | 2015 | RVA/Human-wt/IND/CM-1160/2015/G12P[6] | G12 | P[6] | I1 | R1 | C1 | M1 | A1 | N1 | T1 | E1 | H1 |
| 108 |  | 2016 | RVA/Human-wt/IND/TN030054/2016/G12P[6] | G12 | P[6] | I1 | R1 | C1 | M2 | A1 | N1 | T1 | E1 | H1 |
| 109 | G12P[8] | 2005 | RVA/Human-wt/IND/C-20/2005/G12P[8] | G12 | P[8] | I1 | R1 | C1 | M1 | A1 | N1 | T1 | E1 | H1 |
| 110 |  | 2007 | RVA/Human-wt/IND/C-466/2007/G12P[8] | G12 | P[8] | I1 | R1 | C1 | M1 | A1 | N1 | T1 | E1 | H1 |
| 111 |  | 2008 | RVA/Human-wt/IND/C-764/2008/G12P[8] | G12 | P[8] | I1 | R1 | C1 | M1 | A1 | N1 | T1 | E1 | H1 |
| 112 |  |  | RVA/Human-wt/IND/C-800/2008/G12P[8] | G12 | P[8] | I1 | R1 | C1 | M1 | A1 | N1 | T1 | E1 | H1 |
| 113 |  | 2009 | RVA/Human-wt/IND/C-1057/2009/G12P[8] | G12 | P[8] | I1 | R1 | C1 | M1 | A1 | N1 | T1 | E1 | H1 |
| 114 |  |  | RVA/Human-wt/IND/C-1107/2009/G12P[8] | G12 | P[8] | I1 | R1 | C1 | M1 | A1 | N1 | T1 | E1 | H1 |
| 115 |  | 2012 | RVA/Human-wt/IND/CM-0073/2012/G12P[8] | G12 | P[8] | I1 | R1 | C1 | M1 | A1 | N1 | T1 | E1 | H1 |
| 116 |  |  | RVA/Human-wt/IND/IN1008003_CMC_00044/2012/G12P[8] | G12 | P[8] | I1 | R1 | C1 | M1 | A1 | N1 | T1 | E1 | H1 |
| 117 |  |  | RVA/Human-wt/IND/CM-0100/2012/G12P[8] | G12 | P[8] | I1 | R1 | C1 | M1 | A1 | N1 | T1 | E1 | H1 |
| 118 |  | 2013 | RVA/Human-wt/IND/CM-0204/2013/G12P[8] | G12 | P[8] | I1 | R1 | C1 | M1 | A1 | N1 | T1 | E1 | H1 |
| 119 |  |  | RVA/Human-wt/IND/CM-0211/2013/G12P[8] | G12 | P[8] | I1 | R1 | C1 | M1 | A1 | N1 | T1 | E1 | H1 |
| 120 |  |  | RVA/Human-wt/IND/CM-0225/2013/G12P[8] | G12 | P[8] | I1 | R1 | C1 | M1 | A1 | N1 | T1 | E1 | H1 |
| 121 | G12P[X] | 2011 | RVA/Human-wt/IND/RO1-16475/2011/G12P[x] | G12 | P[x] | I1 | R1 | C1 | Mx | A1 | N1 | T1 | E1 | H1 |
| 122 | G4 | 2011 | RVA/Human-wt/IND/IN1003238_CMC_00038/2011/G4P[6] | G4 | P[6] | I1 | R1 | C1 | M1 | AX | N1 | T1 | E1 | H1 |
| 123 |  | 2010 | RVA/Human-wt/IND/C-1351/2010/G4P[8] | G4 | P[8] | I1 | R1 | C1 | M1 | A1 | N1 | T1 | E1 | H1 |
| 124 | G9P[8] | 2011 | RVA/Human-wt/IND/IN1001096_CMC_00007/2011/G9P[8] | G9 | P[8] | I1 | RX | C1 | M1 | A1 | N1 | T1 | E1 | H1 |
| 125 |  | 2012 | RVA/Human-wt/IND/IN1005022_CMC_00042/2012/G9P[8] | G9 | P[8] | I1 | RX | C1 | M1 | A1 | N1 | T1 | E1 | H1 |
| 126 |  | 2013 | RVA/Human-wt/IND/IN1010229_CMC_00048/2013/G9P[8] | G9 | P[8] | I1 | RX | C1 | M1 | A1 | N1 | T1 | E1 | H1 |
| 127 |  | 2015 | RVA/Human-wt/IND/CM-1261/2015/G9P[8] | G9 | P[8] | I2 | R2 | C2 | Mx | A2 | N2 | T1 | E1 | H2 |

Additional file 3:

Table S3. Whole genome constellation of DS-1-like strains sequenced from Vellore, arranged in the order of year of collection. Green colour box indicates the complete sequence of the classical gene, yellow colour box indicates the complete sequence of the reassortant genes, and the red colour box indicates the missing gene(the segment which could not be sequenced).

|  |  |  |  |  |  | WHOLE GENOME CONSTELLATION | | | | | | | | | | |
| --- | --- | --- | --- | --- | --- | --- | --- | --- | --- | --- | --- | --- | --- | --- | --- | --- |
| **SL.NO** |  | **GENOTYPE** | **YEAR** |  | **SID** | **VP7** | **VP4** | **VP6** | **VP1** | **VP2** | **VP3** | **NSP1** | **NSP2** | **NSP3** | **NSP4** | **NSP5/6** |
| 1 |  | G2P[4] | 2002 |  | RVA/Human-wt/IND/RV-72/2002/G2P[4] | G2 | P[4] | I2 | R2 | C2 | M2 | A2 | N2 | T2 | E2 | H2 |
| 2 |  |  | 2004 |  | RVA/Human-wt/IND/RV-362/2004/G2P[4] | G2 | P[4] | I2 | R2 | C2 | M2 | A2 | N2 | T2 | E2 | H2 |
| 3 |  |  |  |  | RVA/Human-wt/IND/RV-365/2004/G2P[4] | G2 | P[4] | I2 | R2 | C2 | M2 | A2 | N2 | T2 | E2 | H2 |
| 4 |  |  |  |  | RVA/Human-wt/IND/CRI-15462/2004/G2P[4] | G2 | P[4] | I2 | R2 | C2 | M2 | A2 | N2 | T2 | E2 | H2 |
| 5 |  |  |  |  | RVA/Human-wt/IND/CRI-17112/2004/G2P[4] | G2 | P[4] | I2 | R2 | C2 | M2 | A2 | N2 | T2 | E2 | H2 |
| 6 |  |  |  |  | RVA/Human-wt/IND/CRI-21245/2004/G2P[4] | G2 | P[4] | I2 | Rx | C2 | M2 | A2 | N2 | T2 | E2 | H2 |
| 7 |  |  | 2005 |  | RVA/Human-wt/IND/CRI-34630/2005/G2P[4] | G2 | P[4] | I2 | R2 | C2 | M2 | A2 | N2 | T2 | E2 | H2 |
| 8 |  |  |  |  | RVA/Human-wt/IND/C-49/2005/G2P[4] | G2 | P[4] | I2 | R2 | C2 | M2 | A2 | N2 | T2 | E2 | H2 |
| 9 |  |  | 2006 |  | RVA/Human-wt/IND/C-65/2006/G2P[4] | G2 | P[4] | I2 | R2 | C2 | M2 | A2 | N2 | T2 | E2 | H2 |
| 10 |  |  |  |  | RVA/Human-wt/IND/C-421/2006/G2P[4] | G2 | P[4] | I2 | R2 | C2 | M2 | A2 | N2 | T2 | E2 | H2 |
| 11 |  |  |  |  | RVA/Human-wt/IND/C-422/2006/G2P[4] | G2 | P[4] | I2 | R2 | C2 | M2 | A2 | N2 | T2 | E2 | H2 |
| 12 |  |  |  |  | RVA/Human-wt/IND/C-446/2006/G2P[4] | G2 | P[4] | I2 | R2 | C2 | M2 | A2 | N2 | T2 | E2 | H2 |
| 13 |  |  | 2007 |  | RVA/Human-wt/IND/C-471/2007/G2P[4] | G2 | P[4] | I2 | R2 | C2 | M2 | A2 | N2 | T2 | E2 | H2 |
| 14 |  |  |  |  | RVA/Human-wt/IND/C-624/2007/G2P[4] | G2 | P[4] | I2 | R2 | C2 | M2 | A2 | N2 | T2 | E2 | H2 |
| 15 |  |  |  |  | RVA/Human-wt/IND/C-709/2007/G2P[4] | G2 | P[4] | I2 | R2 | C2 | M2 | A2 | N2 | T2 | E2 | H2 |
| 16 |  |  |  |  | RVA/Human-wt/IND/C-724/2007/G2P[4] | G2 | P[4] | I2 | R2 | C2 | M2 | A2 | N2 | T2 | E2 | H2 |
| 17 |  |  | 2008 |  | RVA/Human-wt/IND/C-742/2008/G2P[4] | G2 | P[4] | I2 | R2 | C2 | M2 | A2 | N2 | T2 | E2 | H2 |
| 18 |  |  |  |  | RVA/Human-wt/IND/C-827/2008/G2P[4] | G2 | P[4] | I2 | R2 | C2 | M2 | A2 | N2 | T2 | E2 | H2 |
| 19 |  |  |  |  | RVA/Human-wt/IND/C-839/2008/G2P[4] | G2 | P[4] | I2 | R2 | C2 | M2 | A2 | N2 | T2 | E2 | H2 |
| 20 |  |  |  |  | RVA/Human-wt/IND/C-875/2008/G2P[4] | G2 | P[4] | I2 | R2 | C2 | M2 | A2 | N2 | T2 | E2 | H2 |
| 21 |  |  |  |  | RVA/Human-wt/IND/C-916/2008/G2P[4] | G2 | P[4] | I2 | R2 | C2 | M2 | A2 | N2 | T2 | E2 | H2 |
| 22 |  |  | 2009 |  | RVA/Human-wt/IND/C-1119/2009/G2P[4] | G2 | P[4] | I2 | R2 | C2 | M2 | A2 | N2 | T2 | E2 | H2 |
| 23 |  |  |  |  | RVA/Human-wt/IND/IRID-3271/2009/G2P[4] | G2 | P[4] | I2 | R2 | C2 | M2 | A2 | N2 | T2 | E2 | H2 |
| 24 |  |  |  |  | RVA/Human-wt/IND/IRID-4588/2009/G2P[4] | G2 | P[4] | I2 | R2 | C2 | M2 | A2 | N2 | T2 | E2 | H2 |
| 25 |  |  | 2010 |  | RVA/Human-wt/IND/C-1326/2010/G2P[4] | G2 | P[4] | I2 | R2 | C2 | M2 | A2 | N2 | T2 | E2 | H2 |
| 26 |  |  |  |  | RVA/Human-wt/IND/RO1-2968/2010/G2P[4] | G2 | P[4] | I2 | R2 | C2 | Mx | A2 | N2 | T2 | Ex | H2 |
| 27 |  |  |  |  | RVA/Human-wt/IND/C-1318/2010/G2P[4] | G2 | P[4] | I2 | R2 | C2 | M2 | A2 | N2 | T2 | E2 | H2 |
| 28 |  |  |  |  | RVA/Human-wt/IND/C-1362/2010/G2P[4] | G2 | P[4] | I2 | R2 | C2 | M2 | A2 | N2 | T2 | E2 | H2 |
| 29 |  |  |  |  | RVA/Human-wt/IND/IRID-7766/2010/G2P[4] | G2 | P[4] | I2 | R2 | C2 | M2 | A2 | N2 | T2 | E2 | H2 |
| 30 |  |  |  |  | RVA/Human-wt/IND/RO1-13443/2010/G2P[4] | G2 | P[4] | I2 | R2 | C2 | M2 | A2 | N2 | T2 | E2 | H2 |
| 31 |  |  |  |  | RVA/Human-wt/IND/RO1-14242/2010/G2P[4] | G2 | P[4] | I2 | R2 | C2 | M2 | A2 | N2 | T2 | E2 | H2 |
| 32 |  |  |  |  | RVA/Human-wt/IND/RO1-14688/2010/G2P[4] | G2 | P[4] | I2 | R2 | C2 | M2 | A2 | N2 | T2 | E2 | H2 |
| 33 |  |  | 2011 |  | RVA/Human-wt/IND/IN1001419_CMC_00008/2011/G2P[4] | G2 | P[4] | I2 | R2 | C2 | MX | A2 | N2 | T2 | E2 | H2 |
| 34 |  |  |  |  | RVA/Human-wt/IND/IN1001455_CMC_00009/2011/G2P[4] | G2 | P[4] | I2 | RX | C2 | MX | A2 | N2 | T2 | EX | H2 |
| 35 |  |  |  |  | RVA/Human-wt/IND/LBF-2332/2011/G2P[4] | G2 | P[4] | I2 | R2 | C2 | M2 | A2 | N2 | T2 | E2 | H2 |
| 36 |  |  |  |  | RVA/Human-wt/IND/RO1-22448/2011/G2P[4] | G2 | P[4] | I2 | R2 | C2 | M2 | A2 | N2 | T2 | E2 | H2 |
| 37 |  |  |  |  | RVA/Human-wt/IND/RO1-24270/2011/G2P[4] | G2 | P[4] | I2 | R2 | C2 | M2 | A2 | N2 | T2 | E2 | H2 |
| 38 |  |  |  |  | RVA/Human-wt/IND/IN1003769/2011/G2P[4] | G2 | P[4] | I2 | R2 | C2 | M2 | A2 | N2 | T2 | E2 | H2 |
| 39 |  |  |  |  | RVA/Human-wt/IND/IN1003769_CMC_00016/2011/G2P[4] | G2 | P[4] | I2 | R2 | C2 | M2 | A2 | N2 | T2 | E2 | H2 |
| 40 |  |  | 2012 |  | RVA/Human-wt/IND/RVGE 377/2012/G2P[4] | G2 | P[4] | I2 | R2 | C2 | M2 | A2 | N2 | T2 | E2 | H2 |
| 41 |  |  |  |  | RVA/Human-wt/IND/IN1004030_CMC_00018/2012/G2P[4] | G2 | P[4] | I2 | RX | CX | MX | A2 | N2 | T2 | E2 | H2 |
| 42 |  |  |  |  | RVA/Human-wt/IND/IN1004034_CMC_00019/2012/G2P[4] | G2 | P[4] | I2 | RX | C2 | MX | A2 | N2 | T2 | E2 | H2 |
| 43 |  |  |  |  | RVA/Human-wt/IND/IN1004493_CMC_00040/2012/G2P[4] | G2 | P[4] | I2 | R2 | C2 | M2 | A2 | N2 | T2 | E2 | H2 |
| 44 |  |  |  |  | RVA/Human-wt/IND/IN1004509_CMC_00023/2012/G2P[4] | G2 | P[4] | I2 | RX | C2 | M2 | A2 | N2 | T2 | E2 | H2 |
| 45 |  |  |  |  | RVA/Human-wt/IND/IN1004561_CMC_00024/2012/G2P[4] | G2 | P[4] | I2 | R2 | C2 | M2 | A2 | N2 | T2 | E2 | H2 |
| 46 |  |  |  |  | RVA/Human-wt/IND/IN1004682_CMC_00026/2012/G2P[4] | G2 | P[4] | I2 | RX | C2 | MX | A2 | N2 | T2 | E6 | H2 |
| 47 |  |  |  |  | RVA/Human-wt/IND/LBF-8409/2012/G2P[4] | G2 | P[4] | I2 | R2 | C2 | Mx | A2 | N2 | T2 | E2 | H2 |
| 48 |  |  |  |  | RVA/Human-wt/IND/IN1005331_CMC_00043/2012/G2P[4] | G2 | P[4] | I2 | R2 | C2 | MX | A2 | N2 | T2 | E2 | H2 |
| 49 |  |  |  |  | RVA/Human-wt/IND/CM-0019/2012/G2P[4] | G2 | P[4] | I2 | R2 | C2 | M2 | A2 | N2 | T2 | E2 | H2 |
| 50 |  |  |  |  | RVA/Human-wt/IND/CM-0170/2012/G2P[4] | G2 | P[4] | I2 | R2 | C2 | M2 | A2 | N2 | T2 | E2 | H2 |
| 51 |  |  |  |  | RVA/Human-wt/IND/CM-0002/2012/G2P[4] | G2 | P[4] | I2 | R2 | C2 | M2 | A2 | N2 | T2 | E2 | H2 |
| 52 |  |  |  |  | RVA/Human-wt/IND/CM-0114/2012/G2P[4] | G2 | P[4] | I2 | R2 | C2 | M2 | A2 | N2 | T2 | E2 | H2 |
| 53 |  |  | 2013 |  | RVA/Human-wt/IND/CM-0180/2013/G2P[4] | G2 | P[4] | I2 | R2 | C2 | M2 | A2 | N2 | T2 | E2 | H2 |
| 54 |  |  |  |  | RVA/Human-wt/IND/CM-0286/2013/G2P[4] | G2 | P[4] | I2 | R2 | C2 | M2 | A2 | N2 | T2 | E2 | H2 |
| 55 |  |  |  |  | RVA/Human-wt/IND/CM-0423/2013/G2P[4] | G2 | P[4] | I2 | R2 | C2 | M2 | A2 | N2 | T2 | E2 | H2 |
| 56 |  |  |  |  | RVA/Human-wt/IND/CM-0493/2013/G2P[4] | G2 | P[4] | I2 | R2 | C2 | M2 | A2 | N2 | T2 | E2 | H2 |
| 57 |  |  | 2015 |  | RVA/Human-wt/IND/CM-1146/2015/G2P[4] | G2 | P[4] | I2 | R2 | C2 | M1 | A2 | N2 | T1, T2 | E1,E2 | H1 |
| 58 |  |  |  |  | RVA/Human-wt/IND/CM-1334/2015/G2P[4] | G2 | P[4] | I2 | R2 | C2 | M2 | A2 | N2 | T2 | E2 | H2 |
| 59 |  |  | 2016 |  | RVA/Human-wt/IND/CM-1369/2016/G2P[4] | G2 | P[4] | I2 | R2 | C2 | M2 | A2 | N2 | T2 | E2 | H2 |
| 60 |  |  |  |  | RVA/Human-wt/IND/TN030007/2016/G2P[4] | G2 | P[4] | I2 | R2 | C2 | M2 | A2 | N2 | T2 | E2 | H2 |
| 61 |  |  |  |  | RVA/Human-wt/IND/CM-1536/2016/G2P[4] | G2 | P[4] | I2 | R2 | C2 | M2 | A2 | N2 | T2 | E2 | H2 |
| 62 |  |  |  |  | RVA/Human-wt/IND/TN010329/2016/G2P[4] | G2 | P[4] | I2 | R2 | C2 | M2 | A2 | N2 | T2 | E2 | H2 |
| 63 |  |  |  |  | RVA/Human-wt/IND/TN010382/2016/G2P[4] | G2 | P[4] | I2 | R2 | C2 | M2 | A2 | N2 | T2 | E2 | H2 |
| 64 |  |  |  |  | RVA/Human-wt/IND/TN020143/2016/G2P[4] | G2 | P[4] | I2 | R2 | C2 | M2 | A2 | N2 | T2 | E2 | H2 |
| 65 |  |  | 2017 |  | RVA/Human-wt/IND/TN010437/2017/G2P[4] | G2 | P[4] | I2 | R2 | C2 | M2 | A2 | N2 | T2 | E2 | H2 |
| 66 |  |  |  |  | RVA/Human-wt/IND/TN010476/2017/G2P[4] | G2 | P[4] | I2 | R2 | C2 | M2 | A2 | N2 | T2 | E2 | H2 |
| 67 |  |  |  |  | RVA/Human-wt/IND/TN010491/2017/G2P[4] | G2 | P[4] | I2 | R2 | C2 | M2 | A2 | N2 | T2 | E2 | H2 |
| 68 |  | G2P[4]+P[8] | 2010 |  | RVA/Human-wt/IND/RO1-14518/2010/G2P[4]+P[8] | G2 | P[4]+P[8] | I2 | R2 | C2 | M2 | A2 | N2 | T2, T6 | E2 | H2, H3 |
| 69 |  | G2P[8] | 2012 |  | RVA/Human-wt/IND/IN1004655_CMC_00025/2012/G2P[8] | G2 | P[8] | I2 | RX | C2 | MX | A2 | N2 | T2 | E2 | H2 |
| 70 |  |  |  |  | RVA/Human-wt/IND/IN1005086_CMC_00027/2012/G2P[8] | G2 | P[8] | I2 | R2 | C2 | M2 | A2 | N2 | T2 | E2 | H2 |
| 71 |  | G2P[x] | 2011 |  | RVA/Human-wt/IND/IN1001000_CMC_00004/2011/G2P[x] | G2 | P[X] | I2 | R2 | C2 | M2 | A2 | N2 | T2 | E2 | H2 |
| 72 |  |  |  |  | RVA/Human-wt/IND/LBF-5948/2011/G2P[x] | G2 | P[x] | I2 | Rx | C2 | Mx | A2 | N2 | T1 | E1 | H2 |
| 73 |  | G6 | 2011 |  | RVA/Human-wt/IND/IN1003535_CMC_00014/2011/G6P[x] | G6 | P[X] | I2 | RX | C2 | MX | AX | N2 | T6 | E2 | H3 |
| 74 |  | G9 | 2010 |  | RVA/Human-wt/IND/IN1000951_CMC_00002/2010/G9P[4] | G9 | P[4] | I2 | R2 | C2 | M2 | A2 | N2 | T2 | E2 | H2 |
| 75 |  |  | 2011 |  | RVA/Human-wt/IND/IN1000984_CMC_00003/2011/G9P[4] | G9 | P[4] | I2 | R2 | C2 | M2 | A2 | N2 | T2 | E6 | H2 |
| 76 |  |  |  |  | RVA/Human-wt/IND/IN1001019_CMC_00005/2011/G9P[4] | G9 | P[4] | I2 | R2 | C2 | MX | A2 | N2 | T2 | E6 | H2 |
| 77 |  |  |  |  | RVA/Human-wt/IND/IN1001026_CMC_00006/2011/G9P[4] | G9 | P[4] | I2 | R2 | C2 | MX | A2 | N2 | T2 | E6 | H2 |
| 78 |  |  | 2012 |  | RVA/Human-wt/IND/IN1004802_CMC_00041/2012/G9P[x] | G9 | P[X] | I2 | R2 | C2 | M2 | A2 | N2 | T2 | E2 | H2 |
| 79 |  | P[14] | 2012 |  | RVA/Human-wt/IND/IN1004413_CMC_00022/2012/GxP[14] | GX | P[14] | I2 | R2 | C2 | M2 | AX | N2 | T6 | E2 | H3 |
| 80 |  |  | 2017 |  | RVA/Human-wt/IND/TN020204/2017/G8P[14] | G8 | P[14] | I2 | R2 | C2 | Mx | A11 | N2 | T6 | E2 | H3 |

Additional file 4:

Table S4. Details of the reference strains used for distance analysis and phylogenetic analysis

| S.No | Strain Identifier | Strain Name | Category |
| --- | --- | --- | --- |
| 1 | UFS-1973 | RVA/Human-wt/ZAF/UFS-NGS-MRC-DPRU-1973/2008/G1P[8]I2R2C2M2A2N2T2E2H2 | DS-1 like G1P[8] |
| 2 | UFS-1971 | RVA/Human-wt/ZAF/UFS-NGS-MRC-DPRU-1973/2008/G1P[8]I2R2C2M2A2N2T2E2H2 | DS-1 like G1P[8] |
| 3 | Wa | RVA/Human-wt /USA/WA/1974/G1P[8]I1R1C1M1A1NIT1E1H1 | Human classical |
| 4 | KU | RVA/Human-wt /JAPAN/KU\|1974/G1P[8]I1R1C1M1A1N1T1E1H1 | Human classical |
| 5 | AM06-1 | RVA/Human-wt/IND/AM06-1/2006/G1P[8]I1R1C1M1A1N1T1E1H1 | Human classical |
| 6 | Dhaka16 | RVA/Human-wt/BGD/Dhaka-16/2003/G1P[8]I1R1C1M1A1N1T1E1H1 | Human classical |
| 7 | DS-1 | RVA/Human-wt/USA/DS-1/1976/G2P[4]I2R2C2M2A2N2T2E2H2 | Human classical |
| 8 | 116E3D | RVA/Human-wt/IND/116E3D1993/G2P[4]I2R2C2M2A2N2T2E2H2 | Indian Human classical |
| 9 | Dhaka12 | RVA/Human-wt/BGD/DHAKA12-03/2003/G12P[6]I1R1C1M1A1N1T1E1H1 | Human classical |
| 10 | Matlab13 | RVA/Human-wt/BGD/MATLAB-13/2003/G12P[6]I1R1C1M1A1N1T2E1H1 | Human classical |
| 11 | GER172 | RVA/Human-wt/GER/GER172-08/2008/G12P[6]I1R1C1M1A1N1T1E1H1 | Human classical |
| 12 | GER126 | RVA/Human-wt/GER/GER-126-08/2008/G12P[8]I1R1C1M1A1N1T1E1H1 | Human classical |
| 13 | B4633 | RVA/Human-wt/BEL/B4633/2003/G12P[8]I1R1C1M1A1N1T1E1H1 | Human classical |
| 14 | Dhaka25 | RVA/Human-wt/BGD/DHAKA-25/2002/G12P8I1R1C1M1A1N1T1E1H1 | Human classical |
| 15 | P | RVA/Human-wt/P/1974/G3P[8]I1R1C1M1A1N1T1E1H1 | Human classical |
| 16 | DC2241 | RVA/Human-wt/USA/DC2241/1977/G4P[8]I1R1C1M1A1N1T1E1H1 | Human classical |
| 17 | WI61 | RVA/Human-wt/\|USA/WI61/1975/G9P[8]I1R1C1M1A1N1T1E1H1 | Human classical |
| 18 | ST3 | RVA/Human-wt/GBR/ST3/1975/G4P[6]I1R1C1M1A1N1T1E1H1 | Human classical |
| 19 | RotaTeq-SC2-9 | RVA/Vaccine-tc/USA/RotaTeq-SC2-9/1992/ G2P[5]I2R2C2M1A3N2T6E2H3 | Vaccine |
| 20 | RotaTeq-W179-4 | RVA/Vaccine-tc/USA/RotaTeq-WI79-4/1992/G6P[8]I2R2C2M2A3N2T6E2H3 | Vaccine |
| 21 | RotaTeq-W179-9 | RVA/Vaccine-tc/USA/RotaTeq-WI79-9/1992/G1P5I2R2C2M2A3N2T6E2H3 | Vaccine |
| 22 | 116E | RVA/Vaccine-tc/IND/116E/1985/G9P[11]I1R1C1M1A1N1T1E1H1 | Vaccine |
| 23 | Rotarix | RVA/Vaccine-tc/USA/Rotarix/1988/G1P[8]I1R1C1M1A1N1T1E1H1 | Vaccine |
| 24 | IS1078 | RVA/Human-wt/JPN/IS1078/201/G3P[8]I2R2C2M2A2N2T2E2H2 | Equine like |
| 25 | S13-30 | RVA/Human-wt/JPN/S13-30/2013/G3P[4]I2R2C2M2A2N2T2E2H2 | Equine like |
| 26 | S13-45 | RVA/Human-wt/JPN/S13-45/2013/G2P[4]I2R2C2M2A2N2T2E2H2 | Equine like |
| 27 | BEN-7194 | RVA/Human-wt/\|BEN/3001607194/2016/G9P[4]I2R2C2M2A2N2T2E6H2 | Recent strains |
| 28 | BEN-7196 | RVA/Human-wt/\|BEN/3001607196/2016/G2P[4]I2R2C2M2A2N2T2E2H2 | Recent strains |

Additional file 5:

Table S5. Observed sequence identities among the protein coding regions of the 11 RVA genes among different genotypes of the sequenced Wa-like strains. (NT-Nucleotide identity, AA-Amino Acid identity in percentage).

| **GENE-GENOTYPE** |  | **ALL** | **G1P[8]** | **G12P[8]** | **G12P[6]** |  |
| --- | --- | --- | --- | --- | --- | --- |
|  |  |  |  |  |  |  |
| **VP1 GENE-R1** | NT | 85.41-100 | 93.21-100 | 97.80-99.91 | 94.09-99.97 |  |
|  | AA | 80.10-100 | 88.83-100 | 95.9-99.80 | 90.67-100 |  |
| **VP2 GENE-C1** | NT | 87.62-100 | 96.54-100 | 97.07-99.96 | 93.98-100 |  |
|  | AA | 95.66-100 | 98.19-100 | 98.88-100 | 98.31-100 |  |
| **VP3 GENE-M1** | NT | 87.18-100 | 96.95-100 | 97.66-99.95 | 97.51-100 |  |
|  | AA | 93.24-100 | 97.64-100 | 98.27-100 | 98.43-100 |  |
| **VP6-GENE I1** | NT | 89.26-100 | 95.26-100 | 95.52-100 | 90.69-100 |  |
|  | AA | 97.88-100 | 98.94-100 | 99.20-100 | 98.14-100 |  |
| **VP4-GENE P[8]** | NT | 83.97-100 | 87.28-100 | 96.61-99.96 | - |  |
|  | AA | 83.97-100 | 89.53-100 | 97.81-100 | - |  |
| **VP4-GENE P[6]** | NT | - |  | - | 98.21-100 |  |
|  | AA | - |  | - | 98.74-100 |  |
| **VP7-GENE G1** | NT | 91.94-100 | 91.94-100 | - | - |  |
|  | AA | 92.62-100 | 92.62-100 | - | - |  |
| **VP7-GENE G12** | NT | 95.33-100 | - | 96.24-100 | 95.44-100 |  |
|  | AA | 96.58-100 | - | 97.95-100 | 96.58-100 |  |
| **NSP1 GENE-A1** | NT | 83.74-100 | 95.40-100 | 97.42-100 | 97.65-100 |  |
|  | AA | 81.79-100 | 94.85-100 | 97.94-100 | 97.59-100 |  |
| **NSP2 GENE-N1** | NT | 89.99-100 | 96.55-100 | 96.89-99.89 | 90.55-100 |  |
|  | AA | 93.62-100 | 96.98-100 | 97.99-100 | 93.62-100 |  |
| **NSP3 GENE-T1** | NT | 85.10-100 | 92.49-100 | 92.27-99.78 | 85.87-100 |  |
|  | AA | 90.30-100 | 95.99-100 | 94.98-100 | 90.30-100 |  |
| **NSP4-GENE E1** | NT | 88.14-100 | 93.46-100 | 94.68-100 | 90.80-100 |  |
|  | AA | 91.39-100 | 95.36-100 | 96.03-100 | 93.38-100 |  |
| **NSP5-GENE H1** | NT | 92.77-100 | 97.42-100 | 97.93-100 | 92.94-100 |  |
|  | AA | 91.35-100 | 96.76-100 | 97.30-100 | 91.89-100 |  |

Additional file 6:

Table S6. Observed sequence identities among the protein coding regions of the 11 RVA genes among different genotypes of the sequenced DS-1-like strains. (NT-Nucleotide identity, AA-Amino Acid identity in percentage).

| **GENE-GENOTYPE** |  | **ALL** | **G2P[4]** | **G9P[4]** | **P[14]** |  |
| --- | --- | --- | --- | --- | --- | --- |
|  |  |  |  |  |  |  |
| **VP1 GENE-R2** | NT | 85.13-100 | 85.25-100 | 97.86-100 | 86.62 |  |
|  | AA | 96.29-100 | 97.48-100 | 99.01-100 | 97.48 |  |
| **VP2 GENE-C2** | NT | 83.65-100 | 84.34-100 | 99.16-100 | 98.17 |  |
|  | AA | 94.43-100 | 94.90-100 | 99.30-100 | 99.77 |  |
| **VP3 GENE-M2** | NT | 81.98-100 | 86.26-100 | 98.79-100 | - |  |
|  | AA | 84.62-100 | 89.75-100 | 99.53-100 | - |  |
| **VP6-GENE I2** | NT | 91.62-100 | 97.18-100 | 98.23-100 | 95.81 |  |
|  | AA | 97.77-100 | 98.76-100 | 99.26-100 | 99.26 |  |
| **VP7-GENE G2** | NT | 96.75-100 | 97.10-100 | - | - |  |
|  | AA | 96.85-100 | 97.55-100 | - | - |  |
| **VP7-GENE G9** | NT | 88.95-100 | - | 99.27-100 | - |  |
|  | AA | 91.59-100 | - | 99.35-100 | - |  |
| **VP4 GENE P[4]** | NT | 91.79-100 | 96.73-100 | - | - |  |
|  | AA | 93.27-100 | 98.45-100 | - | - |  |
| **VP4 GENE P[8]** | NT | 83.97-100 | - | - | - |  |
|  | AA | 83.97-100 | - | - | - |  |
| **NSP1 GENE-A2** | NT | 96.92-100 | 96.92-100 | 99.18-100 | - |  |
|  | AA | 95.47-100 | 95.47-100 | 98.56-100 | - |  |
| **NSP2 GENE-N2** | NT | 84.99-100 | 84.99-100 | 99.20-100 | 88.2 |  |
|  | AA | 92.76-100 | 95.17-100 | 98.97-100 | 96.21 |  |
| **NSP3-GENE T2** | NT | 96.71-100 | 97.97-100 | 97.47-100 | - |  |
|  | AA | 98.10-100 | 98.48-100 | 98.10-100 | - |  |
| **NSP3-GENE T6** | NT | 95.32-99.15 | - | - | 95.64 |  |
|  | AA | 98.72-100 | - | - | 98.72 |  |
| **NSP4-GENE E2** | NT | 84.47-100 | 87.88-100 | 91.67 | 93.94 |  |
|  | AA | 90.29-100 | 94.29-100 | 96 | 96.57 |  |
| **NSP4-GENE E6** | NT | 99.42-100 | - | 99.81 | - |  |
|  | AA | 98.82-100 | - | 99.41 | - |  |
| **NSP5-GENE H2** | NT | 97.51-100 | 97.51-100 | 99.00-100 | - |  |
|  | AA | 98.00-100 | 98.00-100 | 99.50-100 | - |  |
| **NSP5-GENE H3** | NT | 96.94-99.78 | - | - | 98.91 |  |
|  | AA | 95.92-100 | - | - | 99.32 |  |

Additional file 7:

Table S7. Observed sequence identities of the protein coding sequences of Wa-like strains compared with reference strains from GenBank. The highest and the lowest identity in comparison with classical strains is highlighted in orange and green respectively. Identity score in comparison to circulating human strains from India are highlighted in red (NT-Nucleotide identity, AA-Amino Acid identity in percentage).

|  | | | **HUMAN CLASSICAL (1970s)** | | | | | | **HUMAN CLASSICAL(2000s)** | | | | | | | |  | **VACCINE** | |
| --- | --- | --- | --- | --- | --- | --- | --- | --- | --- | --- | --- | --- | --- | --- | --- | --- | --- | --- | --- |
| **STRAIN** | | | **Wa** | **KU** | **P** | **DC-2241** | **WI61** | **ST3** | **Dhaka 16** | **AM06-1** | **Dhaka12** | **Matlab13** | **GER172** | **Dhaka25** | **B4633** | **GER126** | **HUMAN STRAINS (INDIA)** | **116E** | **Rotarix** |
| **YEAR/COUNTRY** | | | **1974/USA** | **1974/JPN** | **1974/USA** | **1977/USA** | **1983/USA** | **1975/GBR** | **2003/BGD** | **2006/IND** | **2003/BGD** | **2003/BGD** | **2008/GER** | **2002/BGD** | **2003/BEL** | **2008/GER** |  | **1985/IND** | **1988/USA** |
| **GENE** | **GENOTYPE** | | **G1P[8]** | **G1P[8]** | **G3P[8]** | **G4P[8]** | **G9P[8]** | **G4P[6]** | **G1P[8]** | **G1P[8]** | **G12P[6]** | **G12P[6]** | **G12P[6]** | **G12P[8]** | **G12P[8]** | **G12P[8]** |  | **G9P[11]** | **G1P[8]** |
| **VP1** | **R1** | **NT** | 88.49-98.41 | 85.03-88.67 | 88.49-96.86 | 88.40-98.50 | 88.34-97.21 | 88.40-96.20 | 88.52-99.20 | 88.18-99.20 | 88.34-99.48 | 88.31-98.71 | 88.28-99.79 | 88.52-99.36 | 88.40-99.39 | 88.28-99.54 | 81.79-99.85 | 88.80-96.69 | 88.34-99.48 |
|  |  | **AA** | 93.01-99.45 | 92.56-96.32 | 94.36-99.25 | 94.67-99.26 | 94.58-99.26 | 94.12-98.53 | 94.58-100 | 94.67-99.54 | 94.58-99.72 | 94.67-99.70 | 94.67-100 | 94.58-99.82 | 94.67-99.91 | 94.39-99.63 | 89.35-100 | 94.58-97.25 | 94.58-99.26 |
| **VP2** | **C1** | **NT** | 88.7-94.32 | 88.18-98.88 | 88.55-97.62 | 88.55-94.41 | 88.40-95.96 | 88.03-96.63 | 87.88-99.55 | - | 87.88-99.59 | 88.18-96.91 | 87.84-99.44 | 88.03-96.98 | 87.77-97.13 | 88.44-98.21 | 83.65-100 | 87.69-92.41 | 88.59-94.07 |
|  |  | **AA** | 95.88-98.83 | 96.25-99.44 | 97.07-99.74 | 96.00-98.96 | 96.85-99.22 | 97.06-99.48 | 97.07-99.89 | - | 96.73-99.78 | 96.73-99.49 | 96.96-100 | 96.40-99.35 | 96.51-99.33 | 96.49-99.48 | 88.35-100 | 94.97-98.21 | 96.11-98.83 |
| **VP3** | **M1** | **NT** | 88.80-97.05 | 86.87-94.25 | 87.74-96.59 | 87.74-96.49 | 87.38-94.66 | 87.58-95.78 | 87.74-94.81 | 97.96-99.59 | 87.63-99.49 | 87.53-94.61 | 87.79-99.69 | 87.74-99.69 | 87.53-99.29 | 87.43-99.13 | 84.86-100 | 87.18-88.14 | 88.45-96.28 |
|  |  | **AA** | 95.28-98.11 | 92.61-95.91 | 94.03-98.43 | 94.18-98.27 | 92.92-96.38 | 93.55-97.96 | 94.03-97.64 | 94.18-99.69 | 94.34-100 | 93.71-97.64 | 94.03-99.84 | 94.18-99.84 | 94.18-99.84 | 93.71-99.21 | 87.57-100 | 92..30-94.65 | 94.81-97.64 |
| **VP6** | **I1** | **NT** | 89.34-91.87 | - | 90.30-97.81 | 89.77-98.43 | 90.03-97.55 | 89.60-98.34 | 89-99.56 | 90.03-99.83 | 89.60-99.74 | 89.60-99.74 | 89.34-99.13 | 90.03-99.48 | 89.86-99.48 | 89.77-99.04 | 81.61-100 | 90.12-97.20 | 89.25-91.00 |
|  |  | **AA** | 97.28-98.64 | - | 98.64-99.73 | 98.91-100 | 98.37-99.73 | 98.91-100 | 98.64-100 | 98.91-100 | 98.64-100 | 98.64-100 | 98.64-99.73 | 98.64-100 | 98.91-100 | 98.91-99.73 | 88.42-100 | 97.55-98.91 | 97.55-98.91 |
| **NSP1** | **A1** | **NT** | 84.98-88.34 | 84.19-99.78 | 85.20-90.25 | 85.09-88.23 | 85.09-87.22 | 84.64-95.18 | 84.42-99.44 | 84.30-97.98 | 84.08-99.55 | 84.08-99.55 | 84.08-99.33 | 84.30-99.33 | 84.19-98.09 | 84.75-99.55 | 79.08-100 | 83.18-87.11 | 84.53-90.13 |
|  |  | **AA** | 84.19-87.29 | 84.19-89.35 | 83.85-89 | 84.19-87.29 | 86.94-89.00 | 84.88-93.47 | 82.13-100 | 82.82-97.94 | 82.82-100 | 82.82-100 | 82.13-99.31 | 82.82-100 | 82.13-97.25 | 83.51-99.31 | 79.08-100 | 82.47-88.32 | 83.51-89.35 |
| **NSP2** | **N1** | **NT** | 90.21-92.88 | 88.32-90.21 | 88.54-90.88 | 88.43-91.10 | 89.10-91.99 | 89.43-91.88 | 90.88-99.67 | 90.55-99.44 | 90.32-99.22 | 90.77-99.56 | 90.88-99.78 | 91.10-99.67 | 91.10-99.33 | 90.66-99.22 | 84.45-100 | 91.10-97.00 | 88.99-90.77 |
|  |  | **AA** | 94.63-96.64 | 92.28-94.63 | 95.64-98.32 | 95.30-97.99 | 93.29-95.64 | 93.62-95.97 | 94.97-99.66 | 93.62-99.66 | 94.63-100 | 94.97-99.66 | 94.30-99.66 | 94.97-99.66 | 94.63-100 | 93.96-99.33 | 90.58-100 | 94.30-98.32 | 94.97-96.98 |
| **NSP3** | **T1** | **NT** | 87.75-97.02 | 86.42-95.03 | 87.53-98.12 | 87.86-97.02 | 86.64-96.25 | 87.53-97.90 | 85.76-99.78 | 86.75-99.67 | 85.87-98.90 | - | 85.87-99.78 | 86.64-99.56 | 85.65-99.12 | 87.31-99.67 | 85.01-100 | 86.87-96.47 | 87.09-98.01 |
|  |  | **AA** | 91.64-99.00 | 90.30-96.32 | 92.64-99.00 | 91.97-99.33 | 91.97-96.99 | 92.64-99.33 | 91.64-99.67 | 91.64-99.33 | 91.30-99.00 | - | 91.30-100 | 91.30-99.67 | 91.64-99.33 | 92.31-99.67 | 88.81-100 | 89.63-97.32 | 91.64-98.66 |
| **NSP4** | **E1** | **NT** | 89.57-97.96 | 88.75-95.91 | 89.98-96.32 | 89.57-97.96 | - | 88.75-96.32 | 89.16-99.39 | 89.16-97.34 | 89.37-99.18 | - | 88.96-99.80 | 89.16-99.80 | 88.96-98.57 | 88.96-98.77 | 87.69-100 | 89.37-95.30 | 89.16-97.34 |
|  |  | **AA** | 94.70-98.01 | 92.72-95.36 | 94.70-97.35 | 94.70-98.01 | - | 93.38-96.03 | 92.05-99.34 | 94.70-98.01 | 92.72-100 | - | 92.72-100 | 93.98-100 | 92.05-99.34 | 92.72-99.34 | 85.21-100 | 92.72-97.35 | 92.72-97.35 |
| **NSP5** | **H1** | **NT** | 93.12-94.15 | 92.60-93.80 | 97.93-99.31 | 98.11-99.48 | 97.07-98.11 | 97.25-98.80 | 97.25-99.66 | 97.59-99.31 | 97.93-99.83 | 97.93-99.83 | 97.07-99.31 | 97.93-99.66 | 97.76-100 | 97.42-98.97 | 91.01-100 | 94.66-95.70 | 92.60-93.98 |
|  |  | **AA** | 92.97-95.14 | 92.43-94.59 | 97.30-98.92 | 98.38-100 | 96.22-97.84 | 96.22-98.38 | 96.76-98.92 | 97.30-99.46 | 98.38-100 | 98.38-100 | 98.38-100 | 98.38-100 | 98.38-100 | 98.38-100 | 91.43-100 | 95.68-99.46 | 92.97-95.14 |

Additional file 8:

Table S8. Observed sequence identities of the protein coding sequences of VP4 and VP7 genes of Wa-like strains compared with reference strains from GenBank. The highest and the lowest identity in comparison with reference strains is highlighted in orange and green respectively. (NT-Nucleotide identity, AA-Amino Acid identity in percentage).

| **GENE** | **GENOTYPE** | **HUMAN CLASSICAL(1900s)** | | | | **HUMAN CLASSICAL(2000s)** | | | | | **VACCINE** | | **HUMAN** |
| --- | --- | --- | --- | --- | --- | --- | --- | --- | --- | --- | --- | --- | --- |
| **VP4** | **P8** | **Wa** | **P** | **DC-2241** | **WI61** | **AM06-1** | **Dhaka 16** | **B4633** | **GER 126** | **Dhaka 25** | **ROTATEQ** | **Rotarix** | **IND_HU** |
|  |  | **1974/USA** | **1974/USA** | **1977/USA** | **1983/USA** | **2006/IND** | **2003/BGD** | **2003/BEL** | **2008/GER** | **2002/BGD** | **1992/USA** | **1988/USA** | **ALL/IND** |
|  |  | **G1P[8]** | **G3P[8]** | **G4P[8]** | **G9P[8]** | **G1P[8]** | **G1P[8]** | **G12P[8]** | **G12P[8]** | **G12P[8]** | **G6P[8]** | **G1P[8]** | **P[8]** |
|  |  | 86.86-90.98 | 86.86-96.01 | 87.20-91.15 | 86.81-93.60 | 86.86-98.71 | 86.94-99.29 | 86.84-99.14 | 86.86-99.61 | 87.03-99.53 | 86.81-93.38 | 87.02-91.06 | 83.72-99.96 |
|  |  | 89.31-96.41 | 90.58-97.74 | 89.31-98.20 | 91.10-97.60 | 91.10-98.97 | 91.51-99.100 | 91.48-100 | 91.48-99.61 | 91.61-99.87 | 90.71-96.71 | 89.31-96.99 | 83.97-100 |
|  | **P6** | **Hu Classical (1900s)** | **Hu Classical (2000s)** | | | **HUMAN** |  |  |  |  |  |  |  |
|  |  | **ST3** | **Dhaka 12** | **Matlab 13** | **GER-172** | **IND_HU** |  |  |  |  |  |  |  |
|  |  | **1975/GBR** | **2003/BGD** | **2003/BGD** | **2008/GER** | **ALL/IND** |  |  |  |  |  |  |  |
|  |  | **G4P[6]** | **G12P[6]** | **G12P[6]** | **G12P[6]** | **P[6]** |  |  |  |  |  |  |  |
|  |  | 85.60-94.93 | 85.96-99.29 | 85.78-98.82 | 85.84-99.59 | 82.29-100 |  |  |  |  |  |  |  |
|  |  | 93.61-96.45 | 95.03-99.82 | 95.03-99.82 | 94.85-99.64 | 84.27-100 |  |  |  |  |  |  |  |
| **GENE** | **GENOTYPE** | **Hu CLASSICAL (1900s)** | | **Hu Classical (2000s)** | | **VACCINE** | | **HUMAN** | **G4** | **Hu CLASSICAL(1900s)** | | **HUMAN** |  |
| **VP7** | **G1** | **Wa** | **KU** | **AM06-1** | **Dhaka 16** | **ROTATEQ** | **Rotarix** | **IND_HU** |  | **DC-2241** | **ST3** | **IND_HU** |  |
|  |  | **1974/USA** | **1974/JPN** | **2006/IND** | **2003/BGD** | **1992/USA** | **1988/USA** | **ALL/IND** |  | **1977/USA** | **1975/GBR** | **ALL/IND** |  |
|  |  | **G1P[8]** | **G1P[8]** | **G1P[8]** | **G1P[8]** | **G1P[5]** | **G1P[8]** | **G1** |  | **G4P[8]** | **G4P[6]** | **G4** |  |
|  |  | 91.30-93.14 | 91.50-93.35 | 92.73-99.28 | 92.63-99.28 | 90.89-93.24 | 93.14-97.03 | 86.44-100 |  | 96.43-97.35 | 96.53-97.04 | 85.22-96.64 |  |
|  |  | 93.85-95.69 | 94.46-96.62 | 92.31-99.69 | 93.54-99.38 | 92.92-95.38 | 94.15-97.54 | 84.36-100 |  | 98.47 | 97.55 | 91.90-97.24 |  |
|  | **G12** | **Hu CLASSICAL(2000s)** | | | | | | **HUMAN** | **G9** | **CLASSICAL** | **Recent** | **HUMAN** |  |
|  |  | **Dhaka 12** | **Matlab 13** | **GER-172** | **B4633** | **GER 126** | **Dhaka 25** | **IND_HU** |  | **WI61** | **BEN 7194** | **IND_HU** |  |
|  |  | **2003/BGD** | **2003/BGD** | **2008/GER** | **2003/BEL** | **2008/GER** | **2002/BGD** | **ALL/IND** |  | **1983/USA** | **2016/BEN** | **ALL/IND** |  |
|  |  | **G12P[6]** | **G12P[6]** | **G12P[6]** | **G12P[8]** | **G12P[8]** | **G12P[8]** | **G12** |  | **G9P[8]** | **G9P[4]** | **G9** |  |
|  |  | 96.81-98.75 | 96.70-98.63 | 96.58-99.20 | 96.58-98.97 | 95.33-97.38 | 96.70-99.20 | 91.60-100 |  | 86.13-89.47 | 89.99-99.79 | 88.24-100 |  |
|  |  | 96.92-98.63 | 96.58-98.29 | 98.29-100 | 97.26-98.97 | 94.86-97.60 | 97.60-98.97 | 86.73-100 |  | 89.97-94.82 | 91.59-99.35 | 87.36-100 |  |

Additional file 9:

Table S9. Observed sequence identities of the protein coding sequences of DS-1-like strains compared with reference strains from GenBank. The highest and the lowest identity in comparison with classical strains is highlighted in orange and green respectively. Identity score in comparison to circulating human strains from India are highlighted in red (NT-Nucleotide identity, AA-Amino Acid identity in percentage).

|  | | **HUMAN CLASSICAL** | | **HUMAN RECENT** | | **VACCINE** | | |  |
| --- | --- | --- | --- | --- | --- | --- | --- | --- | --- |
| **STRAIN** | | **DS-1** | **116E3D** | **BEN 7196** | **BEN 7194** | **ROTATEQ-SC2-9** | **ROTATEQ WI79-4** | **ROTATEQ WI79-9** | **HUMAN STRAINS (INDIA)** |
| **YEAR/COUNTRY** | | **1976/USA** | **1993/IND** | **2016/BEN** | **2016/BEN** | **1992/USA** | **1992/USA** | **1992/USA** |  |
| **GENE** | **GENOTYPE** | **G2P[4]** | **G2P[4]** | **G2P[4]** | **G9P[4]** | **G2P[5]** | **G6P[8]** | **G1P[5]** |  |
| **VP1-R2** | **NT** | 86.89-90.94 | 85.72-92.00 | 86.71-97.43 | 86.62-99.48 | 84.01-86.35 | 84.01-86.41 | 84.01-86.35 | **66.67-100** |
|  | **AA** | 96.53-97.89 | 96.53-98.31 | 97.57-99.07 | 97.57-99.63 | 96.04-97.61 | 96.29-97.79 | 96.04-97.61 | **93.72-100** |
| **VP2-C2** | **NT** | 83.93-94.56 | 83.77-97.97 | 84.01-96.83 | 83.36-99.43 | 83.52-93.02 | 83.60-93.10 | 83.60-93.10 | **84.10-100** |
|  | **AA** | 95.07-98.77 | 94.33-98.77 | 95.32-99.75 | 94.33-99.75 | 93.84-98.52 | 94.09-98.77 | 94.09-98.77 | **88.48-100** |
| **VP3-M2** | **NT** | 83.45-93.62 | 82.61-94.77 | 82.78-97.09 | 83.29-99.53 | 75.20-77.60 | 80.56-90.91 | 75.20-77.60 | **82.20-100** |
|  | **AA** | 90.29-96.84 | 89.23-97.22 | 89.45-98.44 | 89.45-98.44 | 80.22-86.17 | 89.81-95.79 | 80.22-86.17 | **88.74-100** |
| **VP6-I2** | **NT** | 85.76-88.32 | 92.13-98.30 | 91.64-98.49 | 91.72-99.50 | 93.73-98.11 | 93.82-98.18 | 93.73-98.11 | **89.96-100** |
|  | **AA** | 98.18-98.74 | 98.63-99.73 | 98.96-99.75 | 98.96-100 | 97.93-99.31 | 97.93-99.31 | 97.93-99.31 | **96.28-100** |
| **NSP1-A2** | **NT** | 91.88-93.39 | 94.76-96.19 | 95.33-97.27 | 97.63-99.71 | - | - | - | **95.00-99.93** |
|  | **AA** | 90.93-93.74 | 94.60-96.98 | 94.17-96.76 | 96.54-99.78 | - | - | - | **91.15-100** |
| **NSP2-N2** | **NT** | 84.08-88.32 | 85.91-98.51 | 86.60-96.79 | 85.34-99.77 | 85.22-88.77 | 85.22-88.77 | 85.22-88.77 | **85.11-100** |
|  | **AA** | 90.69-95.52 | 94.48-99.31 | 93.79-98.62 | 94.48-100 | 91.72-95.86 | 91.72-95.86 | 91.72-95.86 | **92.41-100** |
| **NSP3-T2** | **NT** | 92.70-94.42 | 96.15-98.27 | 96.15-98.14 | 96.95-99.60 | - | - | - | **94.36-100** |
|  | **AA** | 95.60-97.20 | 96.80-98.00 | 97.20-98.80 | 98.40-100 | - | - | - | **94.59-100** |
| **NSP4-E2** | **NT** | 85.07-95.09 | 84.28-97.84 | 83.69-88.80 | - | 85.07-90.37 | 85.07-90.37 | 85.07-90.37 | **82.21-100** |
|  | **AA** | 93.45-97.02 | 93.45-100 | 92.86-97.02 | - | 93.45-95.24 | 93.45-95.24 | 93.45-95.24 | **89.24-100** |
| **NSP5-H2** | **NT** | 94.86-96.02 | - | - | 98.01-98.67 | - | - | - | **97.84-100** |
|  | **AA** | 96.50-97.50 | - | - | 99.00-100 | - | - | - | **97.00-100** |
| **VP7-G2** | **NT** | 92.57-93.84 | 97.1-98.49 | 94.19-95.35 | - | 92.22-93.15 | - | - | **89.15-100** |
|  | **AA** | 94.76-96.15 | 97.55-99.65 | 96.50-98.60 | - | 93.71-95.10 | - | - | **90.79-100** |
| **VP4-P[4]** | **NT** | 90.85-97.46 | 86.60-97.12 | 86.68-96.91 | 87.19-99.51 | - | - | - | **81.58-100** |
|  | **AA** | 91.61-97.03 | 90.45-98.77 | 91.48-98.84 | 91.61-100 | - | - | - | **82.03-100** |
| **VP4-P[14]** | **NT** | - | - | - | - | - | - | - | **94.72-98.51** |
|  | **AA** | - | - | - | - | - | - | - | **96.97-99.66** |
| **NSP1-A11** | **NT** | - | - | - | - | - | - | - | **91.22-94.45** |
|  | **AA** | - | - | - | - | - | - | - | **89.72-95.79** |
| **NSP3-T6** | **NT** | - | - | - | - | 90.64-91.17 | 90.74-91.17 | 90.64-91.17 | **94.57-98.40** |
|  | **AA** | - | - | - | - | 96.15-97.44 | 96.15-97.44 | 96.15-97.44 | **96.47-100** |
| **NSP3-E6** | **NT** | - | - | - | 99.42-99.81 | - | - | - | **96.32-100** |
|  | **AA** | - | - | - | 99.41-100 | - | - | - | **92.94-100** |
| **NSP5-H3** | **NT** | - | - | - | - | 94.75-95.62 | 94.53-95.40 | 94.75-95.62 | **92.56-99.56** |
|  | **AA** | - | - | - | - | 93.88-97.28 | 93.88-97.28 | 93.88-97.28 | **91.16-100** |
